# Three-dimensional descriptors for aminergic GPCRs: dependence on docking conformation and crystal structure

**DOI:** 10.1007/s11030-018-9894-4

**Published:** 2018-11-27

**Authors:** Stanisław Jastrzębski, Igor Sieradzki, Damian Leśniak, Jacek Tabor, Andrzej J. Bojarski, Sabina Podlewska

**Affiliations:** 1 Faculty of Mathematics and Computer Science, Jagiellonian University, S. Łojasiewicza Street 6, 30-048 Kraków, Poland; 2 Department of Medicinal Chemistry, Institute of Pharmacology, Polish Academy of Sciences, Smętna Street 12, 31-343 Kraków, Poland

**Keywords:** Aminergic GPCRs, Docking, Three-dimensional descriptors, Crystal structure, Machine learning

## Abstract

**Abstract:**

Three-dimensional descriptors are often used to search for new biologically active compounds, in both ligand- and structure-based approaches, capturing the spatial orientation of molecules. They frequently constitute an input for machine learning-based predictions of compound activity or quantitative structure–activity relationship modeling; however, the distribution of their values and the accuracy of depicting compound orientations might have an impact on the power of the obtained predictive models. In this study, we analyzed the distribution of three-dimensional descriptors calculated for docking poses of active and inactive compounds for all aminergic G protein-coupled receptors with available crystal structures, focusing on the variation in conformations for different receptors and crystals. We demonstrated that the consistency in compound orientation in the binding site is rather not correlated with the affinity itself, but is more influenced by other factors, such as the number of rotatable bonds and crystal structure used for docking studies. The visualizations of the descriptors distributions were prepared and made available online at http://chem.gmum.net/vischem_stability, which enables the investigation of chemical structures referring to particular data points depicted in the figures. Moreover, the performed analysis can assist in choosing crystal structure for docking studies, helping in selection of conditions providing the best discrimination between active and inactive compounds in machine learning-based experiments.

**Graphical abstract:**

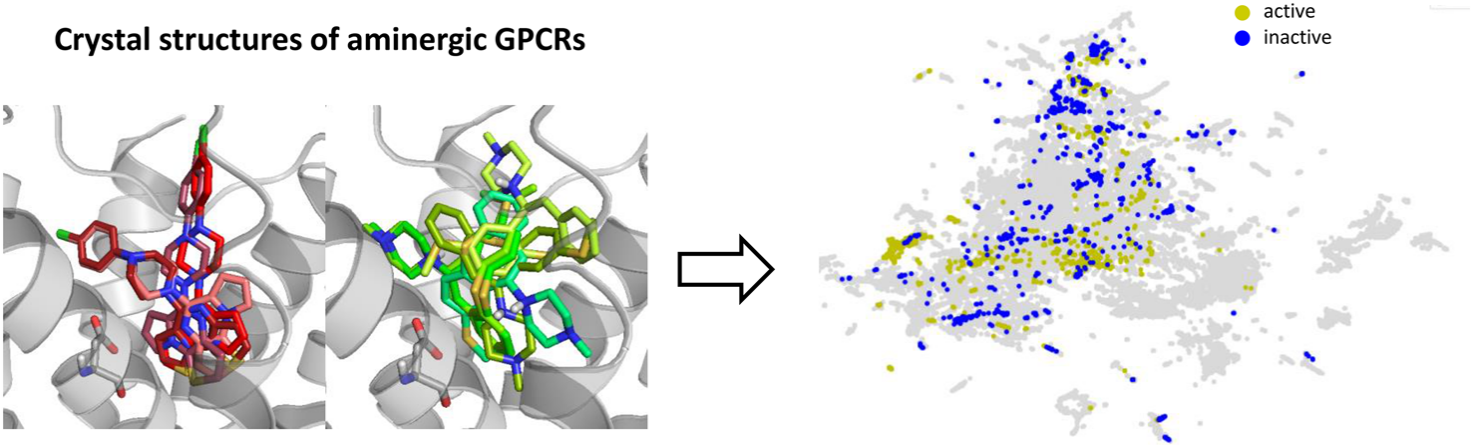

**Electronic supplementary material:**

The online version of this article (10.1007/s11030-018-9894-4) contains supplementary material, which is available to authorized users.

## Introduction

Computational methods are an indispensable part of the drug design process, supporting the search for new compounds with desired biological activity. One of the most popular in silico strategies is virtual screening (VS), in which large libraries of compounds (commercially available or generated computationally using various combinatorial approaches) undergo nonexperimental evaluation [1–3]. Usually, one of the first filters in VS cascades is connected with the application of *ligand*-*based* approaches [4], when only structures of compounds with already determined affinity for a particular receptor are used for making predictions for new compounds. The structures that successfully pass to subsequent filtering steps are typically assessed by the confrontation of the features they possess with pharmacophore models constructed for a particular target [5, 6] and/or are docked to the binding site of the respective protein (*structure*-*based VS* [7]).

One of the fundamental issues connected with the application of various computational methods in the search for new drug candidates is the provision of proper conversion of the chemical structure to a form that can be handled by computer methods. The most popular way to capture chemical information is the application of numerical descriptors or fingerprints [8–11]. The former method usually characterizes the physicochemical properties of compounds, and the examples of such descriptors are as follows: molecular weight, octanol–water partition coefficient (log*P*), *pK*_*a*_, number of hydrogen bond donors, number of hydrogen bond acceptors, number of atoms of a particular type, number of bonds of a particular type, atomic charges, polarity, molecular volume, etc. On the other hand, fingerprints are a result of the translation of compounds into the form of a bit string. Their two main groups can be distinguished: key-based and of a hashed type. Key-based fingerprinting annotates the presence (1) or absence (0) of particular chemical moieties in the molecule, whereas in the hashed fingerprints, bit strings are formed from the molecular graph, on the basis of which paths up to a fixed length are generated (subsequently, starting from each atom), and a hashing function is applied to encapsulate the structural information in the string. Fingerprints are used not only for chemical structure characterization, but they are also applied for description of ligand–receptor complexes obtained in docking. They provide information about the interaction of a ligand with particular amino acids of a protein, as in the case of interaction fingerprints (IFts) [12] and structural interaction fingerprints (SIFts) [13]. The most important advantages of fingerprints are the relative simplicity, low computational costs connected with their generation, and simplicity of making comparisons between two 0–1 strings. The latter procedure can be carried out with the use of various similarity coefficients or by application of machine learning approaches [14, 15].

The *ligand*-*based* approaches usually make also use of various molecular descriptors. They are generated from the two-dimensional (2d) structure of the compound (2d descriptors), or they use their spatial orientation obtained either in minimization of a ligand solely or using the docking poses—three-dimensional (3d) descriptors [16, 17].

Predictive models (constructed most often with the use of machine learning methods of various complexities) based on such representations can be of great help in the search for new active compounds. Such approaches are widely known for the ability to deal with a large amount of information in a fast and efficient way, so the application of machine learning is rapidly growing also in the field of computer-aided drug design, mainly due to a significant increase in amount of data also in the field of cheminformatics and pharmacy. However, despite the great power possessed by various algorithms in the identification of new potentially active compounds, their performance strongly depends on experimental conditions, such as training set compositions, compounds representation, and parameters of particular learning algorithms themselves. Therefore, a multifactor optimization needs to be performed in order to obtain the optimal predictive power of such methods [18, 19].

In this study, we focused on one of the above-mentioned parameters influencing the performance of machine learning methods performance, that is, the compounds representation, 3d descriptors in particular. The variations of their values, depending on the input used for their generation (docking pose), were analyzed. It was examined whether the consistency of 3d descriptor values obtained for different orientations in the binding site of the same compound is correlated with the compound activity. All existing crystal structures of aminergic G protein-coupled receptors (GPCRs) were used for docking of known ligands and compounds with confirmed inactivity toward the respective proteins. The 3d descriptor values calculated for poses obtained from docking for active and inactive compounds were compared with those descriptors that were generated from compounds with minimized energies (prepared in LigPrep for docking) and compared with the analysis of variations in atom positions in the docking poses of a particular compound. The results of such study were also made available online (http://chem.gmum.net/vischem_stability), together with the possibility to manually analyze chemical structures referring to a particular data point. The differences in the distribution of 3d descriptor values indicate that not all crystal structures are effective in this type of experiments (docking followed by automatic analysis of its results), and the prepared tool can be of great help during the design of such studies, especially by assisting in the selection of crystal structures for docking that would provide optimal performance of machine learning methods (when the distribution of descriptor values for active and inactive compounds will be too similar, the machine learning algorithms would also face difficulties in making distinction between these two groups of molecules).

## Methods

The compounds with experimentally determined activity/inactivity were fetched from the ChEMBL database [20] according to the previously described protocol [21]: Only data produced on human- and rat-cloned receptors were considered, sets of active compounds included structures with *K*_*i*_ parameter values below 100 nM (also activities expressed as *IC*_*50*_ were taken into account, assuming that *K*_*i*_ = *IC*_*50*_*/2*) [22], and the sets of inactive compounds included structures with *K*_*i*_ (or equivalent activity parameter) values above 1000 nM. The considered targets were all aminergic GPCRs for which crystal structures are available, including serotonin receptors 5-HT_1B_, 5-HT_2B_, 5-HT_2C_; muscarinic receptors M_1_, M_2_, M_3_, M_4_; adrenergic receptors beta1, beta2; dopamine receptors D_2_, D_3_, D_4_; and histamine receptor H_1_. All respective crystal structures were fetched from the PDB database [23], and they are presented in Table 1.

**Table 1:** Crystal structures used in the study

Target	Crystal structure	Co-crystallized ligand	Resolution ($\AA$)
5-HT_1B_	4IAQ [24]	Dihydroergotamine	2.8
	4IAR [24]	Ergotamine	2.7
	5V54 [25]	CHEMBL428892	3.9
5-HT_2B_	4IB4 [26]	Ergotamine	2.7
	4NC3 [27]	Ergotamine	2.8
	5TUD [28]	Ergotamine	3.0
	5TVN [29]	Lysergide	2.9
5-HT_2C_	6BQG [30]	Ergotamine	3.0
	6BQH [30]	Ritanserin	2.7
ACM_1_	5CXV [31]	CHEMBL258622	2.7
ACM_2_	3UON [32]	(R)-(−)-QNB	3.0
	4MQS [33]	Iperoxo	3.5
	4MQT [33]	LY2119620 Iperoxo	3.7
ACM_3_	4DAJ [33]	Tiotropium	3.4
	4U14 [34]	Tiotropium	3.6
	4U15 [34]	Tiotropium	2.8
	4U16 [34]	Methscopolamine	3.7
ACM_4_	5DSG [35]	Tiotropium	2.6
Beta1	2VT4 [36]	(S)-Cyanopindolol	2.7
	2Y00 [36]	Dobutamine	2.5
	2Y01 [36]	Dobutamine	2.6
	2Y02 [36]	Carmoterol	2.6
	2Y03 [36]	Levisoprenaline	2.9
	2Y04 [36]	Levalbuterol	3.1
	2YCW [37]	Timolol	3.0
	2YCX [37]	(S)-Cyanopindolol	3.3
	2YCY [37]	(S)-Cyanopindolol	3.2
	2YCZ [37]	Timolol	3.7
	3ZPQ [38]	4-(1-Piperazinyl)-1H-indole	2.8
	3ZPR [38]	4-Methyl-2-piperazin-1-yl-quinoline	2.7
	4AMI [39]	Bucindolol	3.2
	4AMJ [39]	(S)-Carvedilol	2.3
	4BVN [40]	(S)-Cyanopindolol	2.1
	4GPO [41]	–	3.5
	5A8E [42]	7-Methylcyanopindolol	2.4
	5F8U [43]	(S)-Cyanopindolol	3.4
Beta2	2R4R [44]	–	3.4
	2R4S [44]	–	3.4
	2RH1 [45]	Carazolol	2.4
	3D4S [46]	Timolol	2.8
	3KJ6 [47]	–	3.4
	3NY8 [48]	AC1NSK5 N	2.8
	3NY9 [48]	CHEMBL1233771	2.8
	3NYA [48]	(−)-Alprenolol	3.2
	3P0G [49]	BI-167107	3.5
	3PDS [50]	FAUC50	3.5
	4GBR [51]	Timolol	4.0
	5D5A [52]	Carazolol	2.5
	5D5B [52]	Carazolol	3.8
	5D6L [53]	Carazolol	3.2
	5X7D [54]	Carazolol PubchemCID: 129318963	2.7
D_2_	6CM4 [55]	Risperidone	2.9
D_3_	3PBL [56]	Eticlopride	2.9
D_4_	5WIU [57]	Nemonapride	2.0
	5WIV [57]	Nemonapride	2.1
H_1_	3RZE [58]	Doxepin	3.1

The compounds were prepared for docking in LigPrep [59] (protonation states generated at pH 7.4 ± 0.0; a maximum of four stereoisomers per compound was allowed), and the number of compounds in each dataset is presented in Table 2.

**Table 2:** Number of compounds from each dataset used in the study

Receptor	Number of compounds before LigPrep		Number of compounds after LigPrep	
	Actives	Inactives	Actives	Inactives
5-HT_1B_	535	435	1174	758
5-HT_2B_	416	311	670	540
5-HT_2C_	1219	1028	1849	1664
ACM_1_	703	964	1335	1000
ACM_2_	727	687	1426	1287
ACM_3_	1054	548	1861	979
ACM_4_	176	352	404	562
Beta1	174	509	264	701
Beta2	230	395	386	556
D_2_	3521	2741	6480	4980
D_3_	2761	799	5203	1272
D_4_	1198	473	1902	827
H_1_	610	587	1301	922

The compounds were docked in Glide to the respective crystal structures; a maximum of ten docking poses were allowed for each compound. For each compound conformation obtained in docking, 3d descriptors were generated using the recently published package for descriptors calculation—Mordred [60]. It contains 214 3d descriptors grouped into the following categories: CPSA, geometrical index, gravitational index, MoRSE, moment of inertia.

**Fig. 1:**
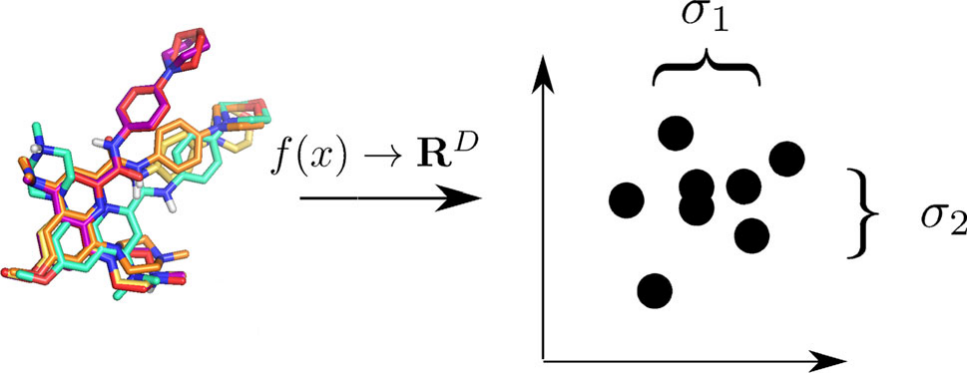
Example analysis of consistency in compounds docked poses presented as the average std of all descriptors used for compounds representation

The descriptor-based compound representations were compared in the following way—for each compound, the standard deviation (std) between values of all descriptors obtained for all docking poses of a particular compound was calculated and visualized using LargeVis by embedding into a 2d space. The results of such analysis were prepared in an interactive way and made available online, so chemical structures referring to a particular data point can be manually analyzed (http://chem.gmum.net/vischem_stability). In addition, studies considering the first *k* conformations (for compound sets sorted by docking score) were performed with *k* adopting values 3, 5, and 10. Moreover, the stds of coordinates of each atom of a compound were calculated (after the compounds alignment) and compared to determine how differences in descriptor values are related to differences in atom positions for various docking poses (Fig. 1).

**Fig. 2:**
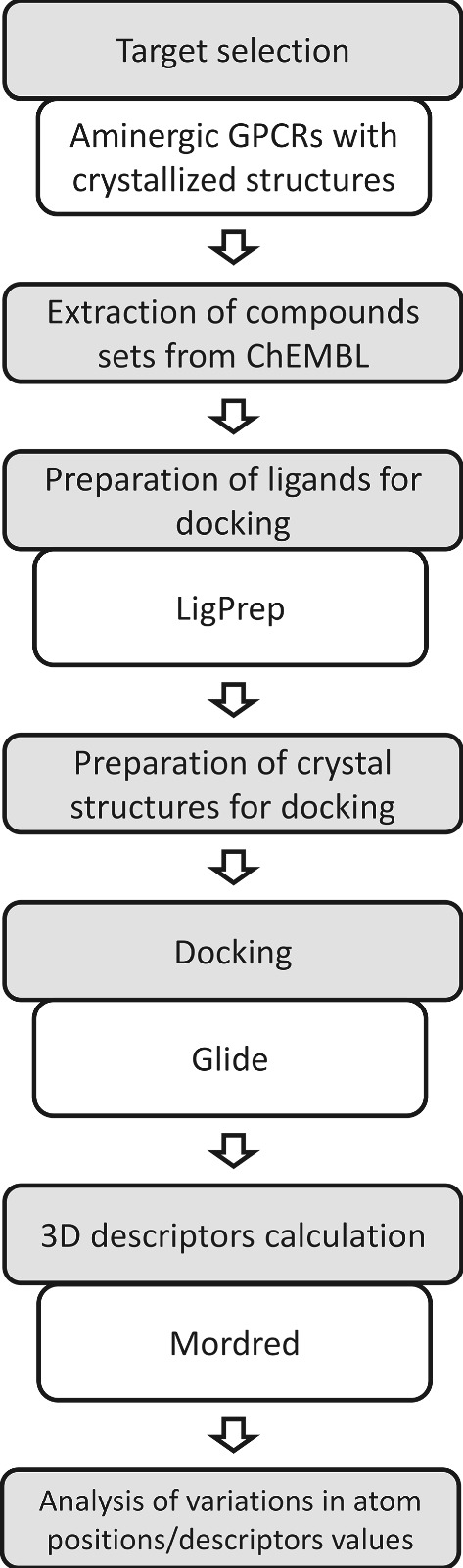
Scheme of the study presented in the paper

The scheme of the whole project is presented in Fig. 2.

## Results and discussion

The averaged std values of generated descriptors between various compound conformations, depending on the number of compound orientations taken into account, are gathered in Table 3 with the stds of atom positions for docking poses obtained for a particular compound.

**Table 3:** Comparison of average std of between analyzed 3d descriptors and atom coordinates

Target	Crystal structure	3d descriptors std/std of atoms positions Number of conformations considered					
		3		5		10	
		Actives	Inactives	Actives	Inactives	Actives	Inactives
5-HT_1B_	4IAQ	**0.254/0.581**	0.208/0.536	**0.309/0.733**	0.252/0.650	**0.332/0.948**	0.271/0.846
	4IAR	**0.259/0.652**	0.212/0.571	**0.312/0.815**	0.260/0.692	**0.335/1.039**	0.278/0.880
	5V54	**0.246/0.582**	0.206/0.535	**0.295/0.719**	0.250/0.668	**0.320/0.890**	0.270/0.884
5-HT_2B_	4IB4	0.191/0.487	**0.219/0.539**	0.243/0.640	**0.266/0.675**	0.258/0.840	**0.281/0.853**
	4NC3	0.194/0.495	**0.217/0.526**	0.242/0.618	**0.272/0.671**	0.257/**0.856**	**0.290/**0.816
	5TUD	0.192/0.486	**0.217/0.524**	0.240/0.614	**0.266/0.656**	0.256/0.792	**0.282/0.807**
	5TVN	0.201/0.478	**0.225/0.529**	0.243/0.602	**0.272/0.657**	0.256/0.758	**0.285/0.838**
5-HT_2C_	6BQG	**0.204/**0.503	0.200/**0.543**	**0.246/**0.627	0.242/**0.666**	**0.258/**0.755	0.257/**0.861**
	6BQH	**0.211/**0.518	0.206/**0.556**	**0.258/**0.650	0.246/**0.672**	**0.272/**0.804	0.262/**0.845**
ACM_1_	5CXV	**0.255/0.572**	0.218/0.539	**0.304/0.716**	0.260/0.670	**0.325/0.907**	0.276/0.854
ACM_2_	3UON	**0.283/0.603**	0.207/0.534	**0.343/0.766**	0.246/0.663	**0.374/0.987**	0.260/0.813
	4MQS	**0.272/0.640**	0.197/0.506	**0.323/0.781**	0.244/0.643	**0.346/1.013**	0.255/0.845
	4MQT	**0.279/0.645**	0.202/0.509	**0.332/0.781**	0.242/0.643	**0.358/1.013**	0.257/0.804
ACM_3_	4DAJ	**0.284/0.587**	0.221/0.523	**0.340/0.736**	0.265/0.647	**0.359/0.998**	0.277/0.821
	4U14	**0.287/0.615**	0.220/0.521	**0.342/0.749**	0.262/0.646	**0.361/0.995**	0.276/0.824
	4U15	**0.288/0.598**	0.224/0.531	**0.343/0.747**	0.270/0.660	**0.362/0.982**	0.282/0.840
	4U16	**0.281/0.563**	0.220/0.533	**0.332/0.706**	0.268/0.671	**0.350/0.957**	0.281/0.844
ACM_4_	5DSG	**0.266/0.597**	0.195/0.506	**0.322/0.774**	0.240/0.626	**0.344/0.969**	0.252/0.797
Beta1	2VT4	0.246/0.555	**0.252/0.557**	0.290/0.675	**0.302/0.683**	0.304/**0.813**	**0.313/**0.792
	2Y00	0.237/0.528	**0.247/0.569**	0.283/0.691	**0.295/0.706**	0.294/0.813	**0.306/0.842**
	2Y01	0.252/**0.624**	**0.255/**0.618	0.299/0.755	**0.309/0.764**	0.312/0.884	**0.320/0.917**
	2Y02	**0.247/0.577**	0.246/0.570	0.293/0.704	**0.299/0.707**	0.306/0.806	**0.313/0.858**
	2Y03	0.252/**0.570**	**0.255/**0.569	0.299/0.698	**0.309/0.711**	0.311/**0.878**	**0.320/**0.838
	2YCW	0.240/0.514	**0.249/0.560**	0.285/0.652	**0.297/0.688**	0.296/0.773	**0.309/0.838**
	2YCX	0.246/**0.547**	**0.248/**0.537	**0.294/0.673**	0.291/0.633	**0.307/**0.786	0.305/**0.830**

Cases where std was higher for active compounds are presented in bold

The analysis clearly shows that the variation of compound orientations in the binding site depends on the crystal structure used for docking rather than on the compound affinity for the receptor. Moreover, the rate of variation in the atom positions is not necessarily correlated with the rate of variation in the 3d descriptor values, although it is the most common situation. Additionally, active compounds were not more consistently docked (in terms of both 3d descriptor values and atom positions) in comparison with inactive molecules, although intuitively and theoretically that should be the case.

The number of docking poses taken into account in the performed analyses has only a slight impact on the observed dependencies that were rather consistent for a particular crystal structure, in terms of both similarity between 3d descriptor values and consistency of compound orientations described as the std of their atom coordinates. For the 5-HT_1B_ crystal structures, the std of both 3d descriptor values and atom coordinates was higher for active than inactive compounds for all cases. On the other hand, 5-HT_2B_ ligands were, in general, more consistent in terms of the analyzed properties than compounds that were inactive for this receptor.

Interestingly, 5-HT_2C_ and ACM_1_ ligands were more consistent compared with inactive compounds in terms of atom coordinates but less consistent when 3d descriptors were considered. However, for the rest of the muscarinic receptors, the tendencies were again shifted toward higher inconsistency for active compounds.

Adrenergic receptors were, in general, characterized by higher consistency for active compounds in terms of both parameters considered; however, there are examples of crystal structures for which there were variations in the consistency of 3d descriptor values and atom coordinates—such as 2R4R, 2R4S, 3KJ6, 3P0G, and 5D5A. The above-mentioned differences occurred also for the crystal structure of histamine receptor H_1_.

**Fig. 3:**
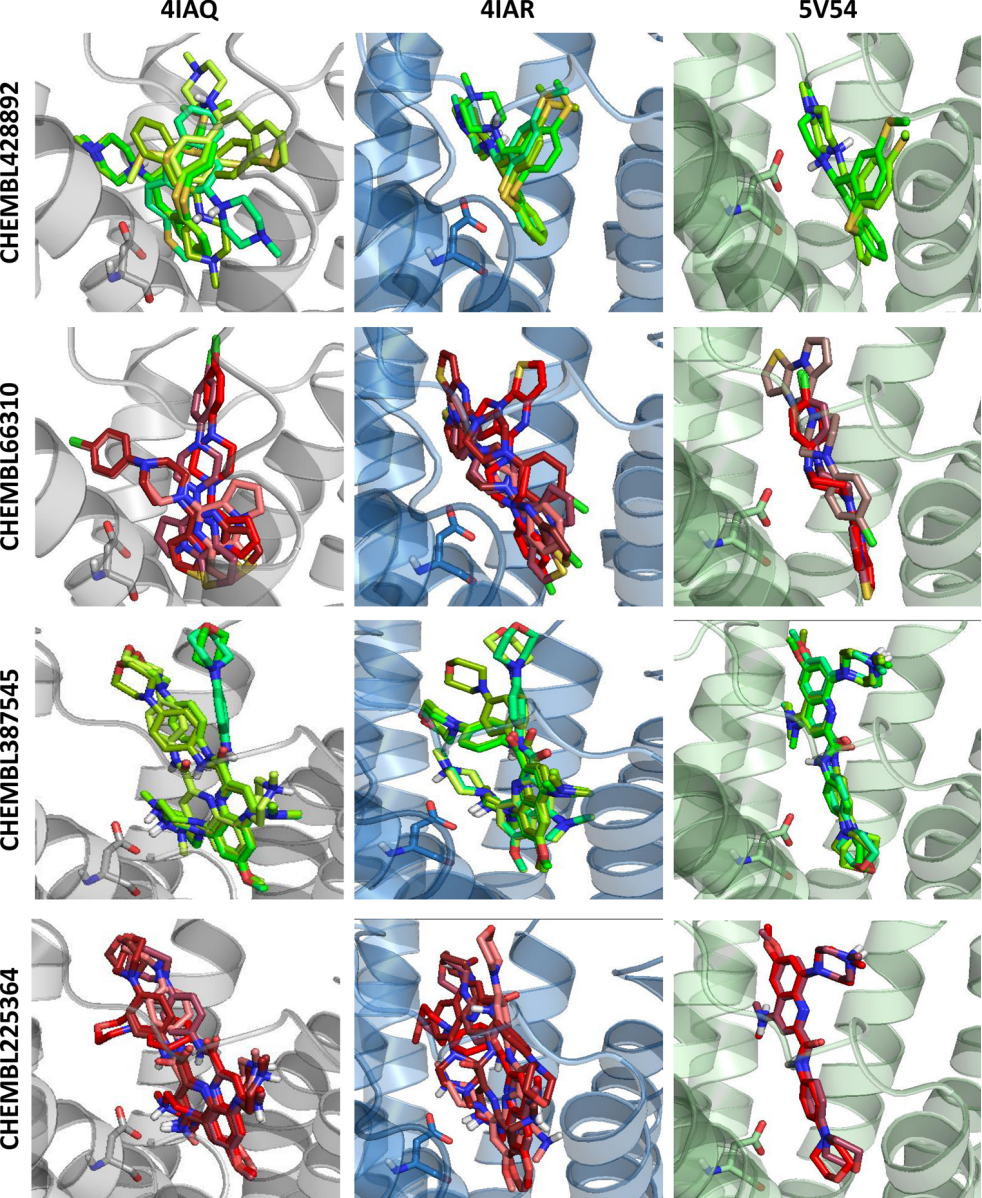
Analysis of docking results of selected compounds to crystal structures of serotonin receptor 5-HT_1B_; green: active compound, red: inactive compound; D3.32 residue of the protein is visualized as sticks. (Color figure online)

Example differences in docking poses for various crystal structures for two pairs of structurally similar active and inactive compounds (CHEMBL428892, CHEMBL66310, and CHEMBL387545, CHEMBL225364 pairs) toward 5-HT_1B_ are presented in Fig. 3.

The docking results presented in Fig. 3 confirm the strong dependence of the consistency of a compound docking pose on the crystal structure used for docking. The active compound, CHEMBL428892, was docked in varying poses for the 4IAQ crystal structure, whereas when 4IAR and 5V54 were used, it similarly fit in the respective binding site. On the other hand, its inactive analog, CHEMBL66310, was docked less consistently to all crystals used; the highest variations occurred for 5V54, as one of the poses was flipped. CHEMBL387545 adopted two different orientations in the binding site of 4IAQ, and two in 4IAR, whereas for 5V54, all of the obtained docking poses were similar. CHEMBL225364, despite being inactive toward 5-HT_1B_, was docked very similarly to its 4IAQ and 5V54 crystal structures with conformation variations occurring for 4IAR.

**Fig. 4:**
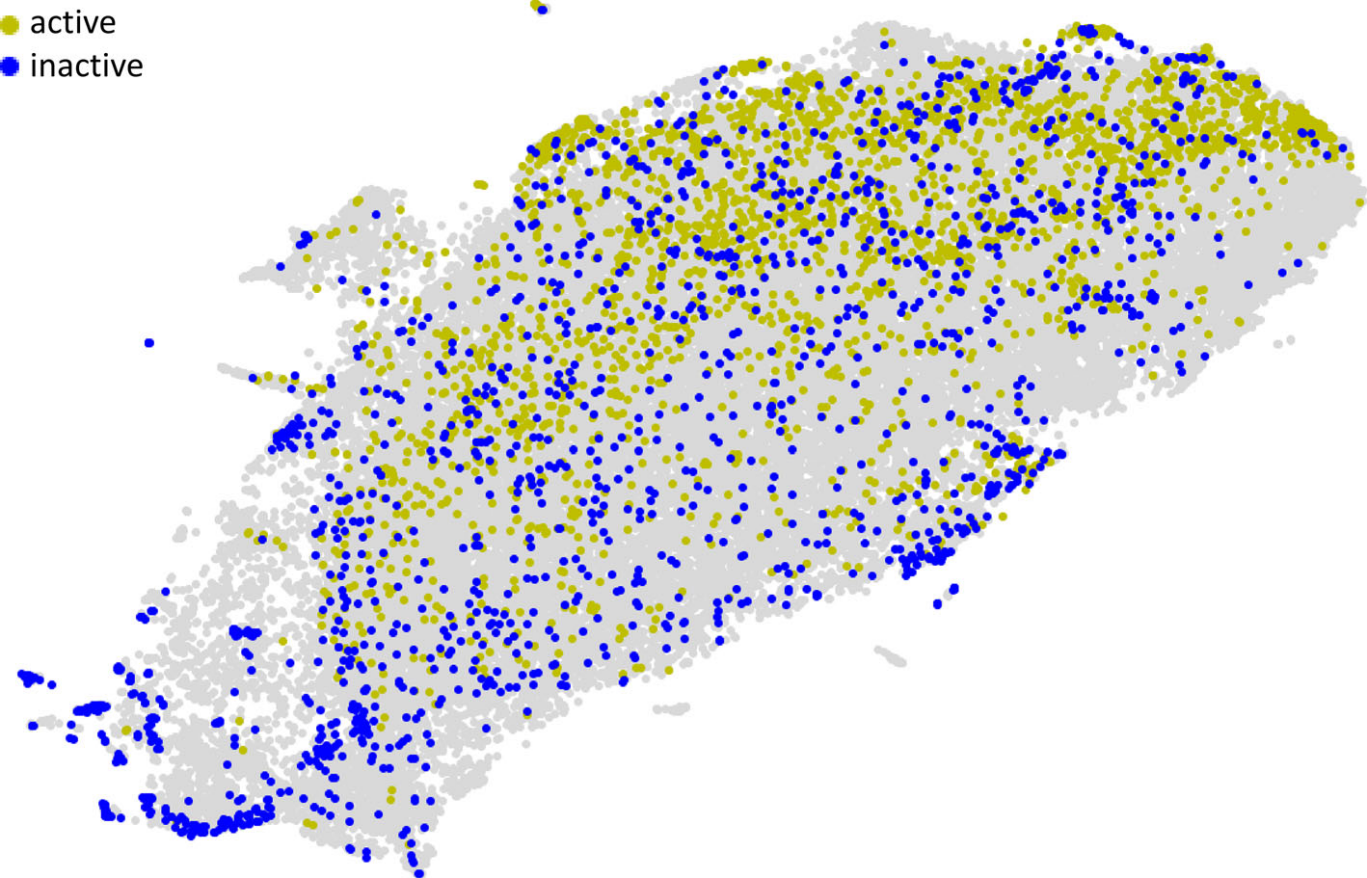
Example analysis of stability of docking poses for ligands of the ACM_3_ receptor. Gray area refers to compounds active or inactive toward other targets considered in the study. In this case, active compounds are relatively well demarcated from the inactive ones

Example visualization of the 3d descriptors space is presented in Fig. 4. It can be observed that for the outcome of this docking studies, representation by 3d descriptors will not necessarily provide good performance of predictive model constructed with the aim of making distinction between active and inactive compounds toward ACM_3_.

**Fig. 5:**
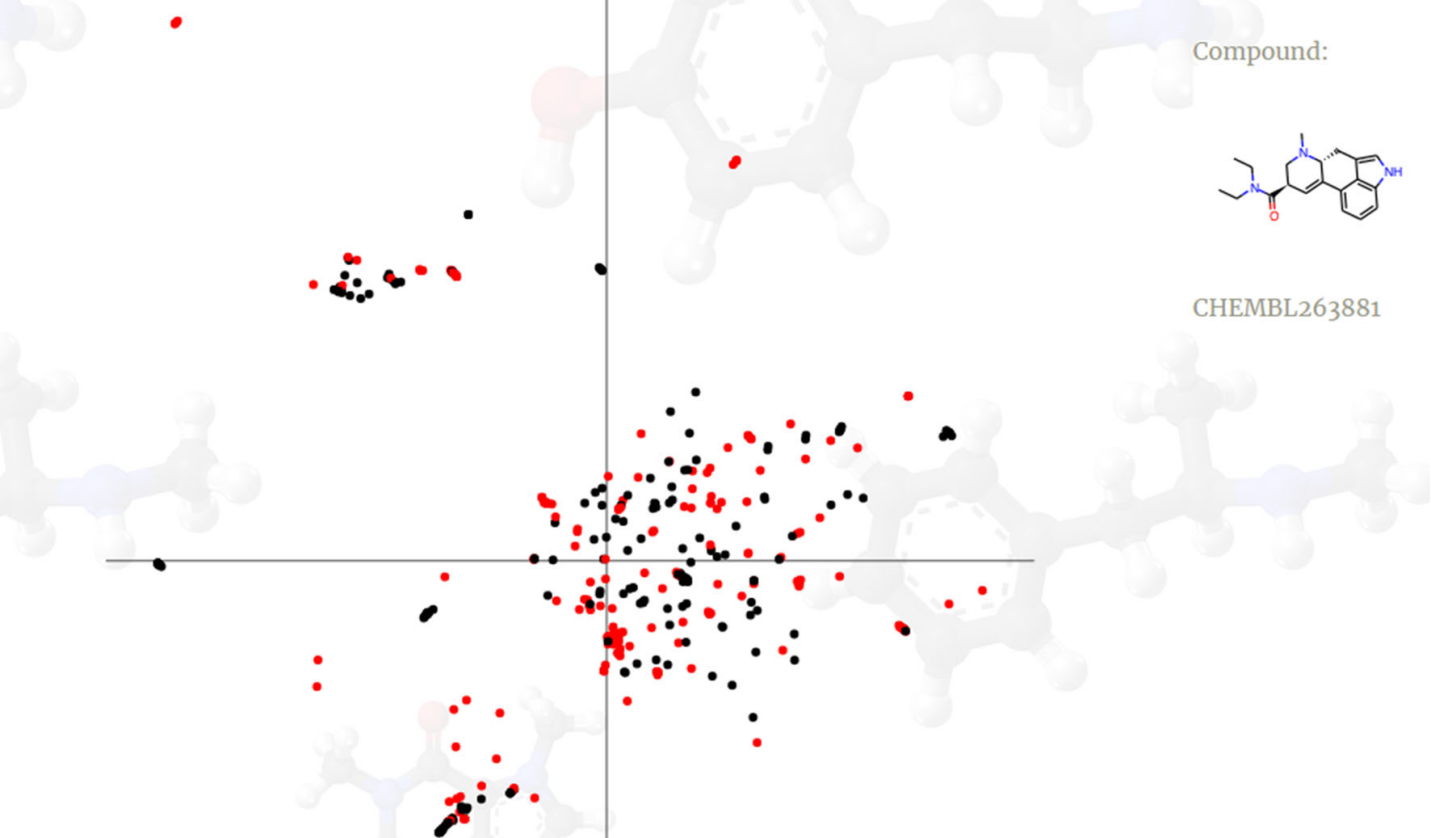
Example visualization of the prepared online tool. Red points refer to active compounds and black to the inactive ones. Chemical structure corresponding to each data point can be manually analyzed. (Color figure online)

Visualizations for all crystal structures are available online at http://chem.gmum.net/vischem_stability, together with the provision of compound structures referring to a particular data point (Fig. 5). The possibility to manually analyze the results enables the explanation and optimization of machine learning methods performance and other automatic and semiautomatic analyses performed on 3d descriptors as compounds representation. Such visualizations can be of great help at the stage of designing such experiments, e.g., in terms of choosing the crystal structure that provides the best discrimination between active and inactive compounds, especially considering that the consistency in 3d descriptor space is not necessarily correlated with the std of atom coordinates in various docking poses, or in terms of proper evaluation of the applicability of a model, by the analysis of chemical structures of compounds that occupy similar regions of 3d descriptors space, despite possessing different activities toward considered receptor.

**Fig. 6:**
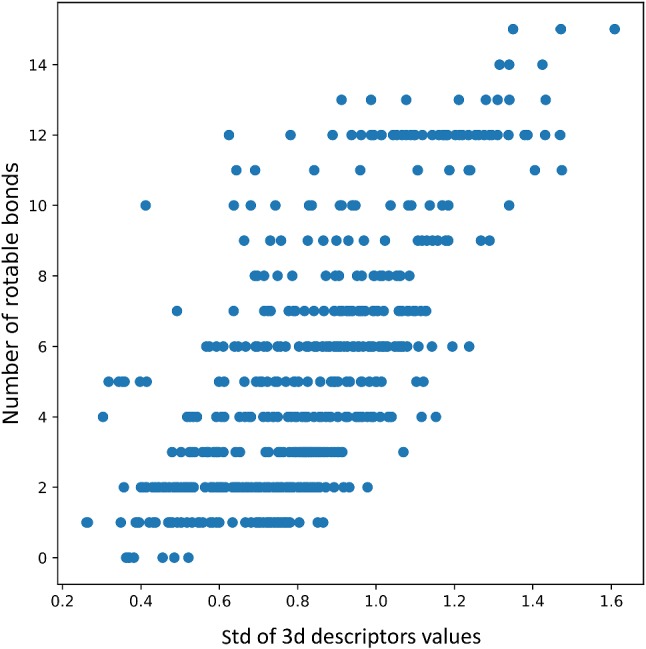
Analysis of average std of the 3d descriptor values depending on the number of rotatable bonds in a particular compound for 5-HT_2B_R ligands, when ten docked poses were taken into account

To explain the causes of such dependencies of the compound pose variations, the analysis of the relationship between the number of rotatable bonds and the std in 3d descriptor values, expressed as the Pearson correlation coefficient, was carried out (Table 4; for simplification, the results were averaged for all crystal structures for a particular protein). According to intuition, the performed analysis indicated a strong dependency of the consistency in descriptor values on the number of rotatable bonds (the higher the number of rotatable bonds, the lower the consistency of 3d descriptor values) with Pearson correlation values approaching 0.718 for 5-HT_2B_. Another observation is that in general, the correlation between the number of rotatable bonds and the variations in compound orientations in the binding site (described by 3d descriptors) was stronger when a higher number of docked poses was taken into account. Example visualizations of such dependencies are presented in Fig. 6, and respective figures for the remaining data are present in the Supplementary Material.

**Table 4:** Comparison of values of the Pearson correlation coefficient between the number of rotatable bonds and the average std of the 3d descriptor values for particular compound conformations

Target	Number of compound conformations considered/Pearson correlation coefficient value		
	3	5	10
5-HT_1B_	0.367	0.452	0.546
5-HT_2B_	0.506	0.562	0.718
5-HT_2C_	0.442	0.519	0.618
ACM_1_	0.247	0.326	0.513
ACM_2_	0.289	0.316	0.562
ACM_3_	0.285	0.330	0.562
ACM_4_	0.265	0.462	0.657
Beta1	0.290	0.329	0.446
Beta2	0.185	0.211	0.332
D_2_	0.345	0.422	0.595
D_3_	0.283	0.334	0.530
D_4_	0.309	0.375	0.560
H_1_	0.190	0.218	0.000

Moreover, an analysis of std of each descriptor values separately was performed (Supplementary Material; for simplification, the obtained std values were averaged for all crystal structures available for a particular protein). It was found out that the lowest variation occurred for 3D-MoRSE descriptors determined for a distance equal to 1 (std of 1–2 × 10^−18^), whereas the highest std values were on average obtained for descriptors from the group of geometrical indexes, such as the geometric Petitjean index and the geometrical shape index (std of 0.5–0.7 on average). Relatively high fluctuations in descriptor values (of similar std range as in the case of the geometrical shape index descriptors) were also observed for 3D-MoRSE descriptors calculated for a distance equal to 10.

## Concluding remarks

In summary, a deep analysis of 3d descriptors generated for compounds docked to crystal structures of aminergic GPCRs, with the aim of their application in machine learning experiments, supplemented with the tool for manual inspection of structures referring to a particular data point was prepared. A strong dependence of the obtained results on the crystal structure used for docking was proved. Moreover, although the variation of 3d descriptor values is typically correlated with the variation of compound conformations, there were several cases where these dependencies were not preserved, revealing the limitations of the applied depiction of docking poses (for the ideal representation, higher variation in docking poses should led to higher variation in descriptor values and vice versa). Additionally, the ability to manually analyze all the information, including the analysis of chemical structures referring to particular data points, enables a better design of machine learning experiments conducted on such type of data, allowing for the maximization of the power of predictive models, for example by proper selection of crystal structures for studies (those that provide the best discrimination between active and inactive compounds in machine learning-based experiments) or the proper evaluation of the model applicability via the analysis of chemical structures with overlapping fragments of descriptors space.

## Electronic supplementary material

Below is the link to the electronic supplementary material.

Supplementary File S1 Analysis of the std of descriptor values calculated for each descriptor separately (XLSX 80 kb)

Supplementary File S2 Visualization of the correlation between the number of rotatable bonds and the variations in atom positions in the docked poses (ZIP 1316 kb)

## References

[1] Reddy AS, Pati SP, Kumar PP, Pradeep HN, Sastry GN (2007). Virtual screening in drug discovery—a computational perspective. Curr Prot Pept Sci.

[2] Breda A, Basso LA, Santos DS, de Azevedo WF (2008). Virtual screening of drugs: score functions, docking, and drug design. Curr Comput Aided Drug Des.

[3] Nicholls A (2008). What do we know and when do we know it?. J Comput Aided Mol Des.

[4] Geppert H, Vogt M, Bajorath J (2010). Current trends in ligand-based virtual screening: molecular representations, data mining methods, new application areas, and performance evaluation. J Chem Inf Model.

[5] Gao Q, Yang L, Zhu Y (2010). Pharmacophore based drug design approach as a practical process in drug discovery. Curr Comput Aided Drug Des.

[6] Yang SY (2010). Pharmacophore modeling and applications in drug discovery: challenges and recent advances. Drug Discov Today.

[7] Anderson AC (2003). The process of structure-based drug design. Chem Biol.

[8] Heikamp K, Bajorath J (2011). How do 2D fingerprints detect structurally diverse active compounds? Revealing compound subset-specific fingerprint features through systematic selection. J Chem Inf Model.

[9] Duan J, Sastry M, Dixon S, Lowrie J, Sherman W (2010). Analysis and comparison of 2D fingerprints: insights into database screening performance using eight fingerprint methods. J Mol Gr Model.

[10] Clark RD, Patterson DE, Soltanshahi F, Blake JF, Matthew JB (2000). Visualizing substructural fingerprints. J Mol Gr Model.

[11] Hert J, Willett P, Wilton DJ, Acklin P, Azzaoui K, Jacoby E, Schuffenhauer A (2004). Comparison of topological descriptors for similarity-based virtual screening using multiple bioactive reference structures. Org Biomol Chem.

[12] Marcou G, Rognan D (2007). Optimizing fragment and scaffold docking by use of molecular interaction fingerprints. J Chem Inf Model.

[13] Deng Z, Chuaqui C, Singh J (2006). Knowledge-based design of target-focused libraries using protein–ligand interaction constraints. J Med Chem.

[14] Bender A, Jenkins JL, Scheiber J, Sukuru SCK, Glick M, Davies J (2009). W. How similar are similarity searching methods? A principal component analysis of molecular descriptor space. J Chem Inf Model.

[15] Willett P, Barnard JM, Downs GM, Information BC, Road U, Sheffield S (1998). Chemical similarity searching. J Chem Inf Comput Sci.

[16] Acharya C, Coop A, Polli JE, MacKerell AD (2011). Recent advances in ligand-based drug design: relevance and utility of the conformationally sampled pharmacophore approach. Curr Comput Aided Drug Des.

[17] Lounnas V, Ritschel T, Kelder J, McGuire R, Bywater RP, Foloppe N (2013). Current progress in structure-based rational drug design marks a new mindset in drug discovery. Comput Struct Biotechnol J.

[18] Mitchell JBO (2014). Machine learning methods in chemoinformatics. Wiley Interdiscip Rev Comput Mol Sci.

[19] Melville JL, Burke EK, Hirst JD (2009). Machine learning in virtual screening. Comb Chem High Throughput Screen.

[20] Gaulton A, Bellis LJ, Bento AP, Chambers J, Davies M, Hersey A, Light Y, McGlinchey S, Michalovich D, Al-Lazikani B, Overington JP (2011). ChEMBL: a large-scale bioactivity database for drug discovery. Nucleic Acids Res.

[21] Warszycki D, Mordalski S, Kristiansen K, Kafel R, Sylte I, Chilmonczyk Z, Bojarski AJ (2013). A linear combination of pharmacophore hypotheses as a new tool in search of new active compounds—an application for 5-HT1A receptor ligands. PLoS ONE.

[22] Kalliokoski T, Kramer C, Vulpetti A, Gedeck P (2013). Comparability of mixed IC50 data—a statistical analysis. PLoS ONE.

[23] Berman HM, Westbrook J, Feng Z, Gilliland G, Bhat TN, Weissig H, Shindyalov IN, Bourne PE (2000). The Protein Data Bank. Nucleic Acids Res.

[24] Wang C, Jiang Y, Ma J, Wu H, Wacker D, Katritch V, Han GW, Liu W, Huang X-P, Vardy E, McCorvy JD, Gao X, Zhou XE, Melcher K, Zhang C, Bai F, Yang H, Yang L, Jiang H, Roth BL, Cherezov V, Stevens RC, Xu HE (2013). Structural basis for molecular recognition at serotonin receptors. Science.

[25] Yin WC, Zhou XE, Yang D, De Waal P, Wang MT, Dai A, Cai X, Huang CY, Liu P, Yin Y, Liu B, Caffrey M, Melcher K, Xu Y, Wang MW, Xu HE, Jiang Y (2018). Crystal structure of the human 5-HT1B serotonin receptor bound to an inverse agonist. Cell Discov.

[26] Wacker D, Wang C, Katritch V, Han GW, Huang X-P, Vardy E, McCorvy JD, Jiang Y, Chu M, Siu FY, Liu W, Xu HE, Cherezov V, Roth BL, Stevens RC (2013). Structural features for functional selectivity at serotonin receptors. Science.

[27] Liu W, Wacker D, Gati C, Han GW, James D, Wang D, Nelson G, Weierstall U, Katritch V, Barty A, Zatsepin NA, Li D, Messerschmidt M, Boutet S, Williams GJ, Koglin JE, Seibert MM, Wang C, Shah STA, Basu S, Fromme R, Kupitz C, Rendek KN, Grotjohann I, Fromme P, Kirian RA, Beyerlein KR, White TA, Chapman HN, Caffrey M, Spence JCH, Stevens RC, Cherezov V (2013). Serial femtosecond crystallography of G protein-coupled receptors. Science.

[28] Ishchenko A, Wacker D, Kapoor M, Zhang A, Han GW, Basu S, Patel N, Messerschmidt M, Weierstall U, Liu W, Katritch V, Roth BL, Stevens RC, Cherezov V (2017). Structural insights into the extracellular recognition of the human serotonin 2B receptor by an antibody. Proc Natl Acad Sci.

[29] Wacker D, Wang S, Mccorvy JD, Betz RM, Venkatakrishnan AJ, Levit A, Lansu K, Schools ZL, Che T, Nichols DE, Shoichet BK, Dror RO, Roth BL (2017). Crystal structure of an LSD-bound human serotonin receptor. Cell.

[30] Peng Y, McCorvy JD, Harpsøe K, Lansu K, Yuan S, Popov P, Qu L, Pu M, Che T, Nikolajsen LF, Huang XP, Wu Y, Shen L, Bjørn-Yoshimoto WE, Ding K, Wacker D, Han GW, Cheng J, Katritch V, Jensen AA, Hanson MA, Zhao S, Gloriam DE, Roth BL, Stevens RC, Liu ZJ (2018). 5-HT2C receptor structures reveal the structural basis of GPCR polypharmacology. Cell.

[31] Thal DM, Sun B, Feng D, Nawaratne V, Leach K, Felder CC, Bures MG, Evans DA, Weis WI, Bachhawat P, Kobilka TS, Sexton PM, Kobilka BK, Christopoulos A (2016). Crystal structures of the M1 and M4 muscarinic acetylcholine receptors. Nature.

[32] Haga K, Kruse AC, Asada H, Yurugi-Kobayashi T, Shiroishi M, Zhang C, Weis WI, Okada T, Kobilka BK, Haga T, Kobayashi T (2012). Structure of the human M2 muscarinic acetylcholine receptor bound to an antagonist. Nature.

[33] Kruse AC, Ring AM, Manglik A, Hu J, Hu K, Eitel K, Hübner H, Pardon E, Valant C, Sexton PM, Christopoulos A, Felder CC, Gmeiner P, Steyaert J, Weis WI, Garcia KC, Wess J, Kobilka BK (2013). Activation and allosteric modulation of a muscarinic acetylcholine receptor. Nature.

[34] Kruse AC, Hu J, Pan AC, Arlow DH, Rosenbaum DM, Rosemond E, Green HF, Liu T, Chae PS, Dror RO, Shaw DE, Weis WI, Wess J, Kobilka BK (2012). Structure and dynamics of the M3 muscarinic acetylcholine receptor. Nature.

[35] Thorsen TS, Matt R, Weis WI, Kobilka BK (2014). Modified T4 lysozyme fusion proteins facilitate G protein-coupled receptor crystallogenesis. Structure.

[36] Warne T, Serrano-Vega MJ, Baker JG, Moukhametzianov R, Edwards PC, Henderson R, Leslie AGW, Tate CG, Schertler GFX (2008). Structure of a β1-adrenergic G-protein-coupled receptor. Nature.

[37] Moukhametzianov R, Warne T, Edwards PC, Serrano-Vega MJ, Leslie AGW, Tate CG, Schertler GFX (2011). Two distinct conformations of helix 6 observed in antagonist-bound structures of a β1-adrenergic receptor. Proc Natl Acad Sci.

[38] Christopher JA, Brown J, Doré AS, Errey JC, Koglin M, Marshall FH, Myszka DG, Rich RL, Tate CG, Tehan B, Warne T, Congreve M (2013). Biophysical fragment screening of the β1-adrenergic receptor: identification of high affinity arylpiperazine leads using structure-based drug design. J Med Chem.

[39] Warne T, Edwards PC, Leslie AGW, Tate CG (2012). Crystal structures of a stabilized β1-adrenoceptor bound to the biased agonists bucindolol and carvedilol. Structure.

[40] Miller-Gallacher JL, Nehmé R, Warne T, Edwards PC, Schertler GFX, Leslie AGW, Tate CG (2014). The 2.1 Å resolution structure of cyanopindolol-bound β1-adrenoceptor identifies an intramembrane Na + ion that stabilises the ligand-free receptor. PloS ONE.

[41] Huang J, Chen S, Zhang JJ, Huang XY (2013). Crystal structure of oligomeric β1-adrenergic G protein-coupled receptors in ligand-free basal state. Nat Struct Mol Biol.

[42] Sato T, Baker J, Warne T, Brown GA, Leslie AGW, Congreve M, Tate CG (2015). Pharmacological analysis and structure determination of 7-methylcyanopindolol-bound β1-adrenergic receptor. Mol Pharmacol.

[43] Leslie AGW, Warne T, Tate CG (2015). Ligand occupancy in crystal structure of β1-adrenergic G protein-coupled receptor. Nat Struct Mol Biol.

[44] Rasmussen SGF, Choi H-J, Rosenbaum DM, Kobilka TS, Thian FS, Edwards PC, Burghammer M, Ratnala VRP, Sanishvili R, Fischetti RF, Schertler GFX, Weis WI, Kobilka BK (2007). Crystal structure of the human beta2 adrenergic G-protein-coupled receptor. Nature.

[45] Cherezov V, Rosenbaum DM, Hanson MA, Rasmussen SGF, Thian FS, Kobilka TS, Choi H-J, Kuhn P, Weis WI, Kobilka BK, Stevens RC (2007). High-resolution crystal structure of an engineered human beta2-adrenergic G protein-coupled receptor. Science.

[46] Hanson MA, Cherezov V, Griffith MT, Roth CB, Jaakola VP, Chien EYT, Velasquez J, Kuhn P, Stevens RC (2008). A specific cholesterol binding site is established by the 2.8 Å structure of the human beta2-adrenergic receptor. Structure.

[47] Bokoch MP, Zou Y, Rasmussen SGF, Liu CW, Nygaard R, Rosenbaum DM, Fung JJ, Choi HJ, Thian FS, Kobilka TS, Puglisi JD, Weis WI, Pardo L, Prosser RS, Mueller L, Kobilka BK (2010). Ligand-specific regulation of the extracellular surface of a G-protein-coupled receptor. Nature.

[48] Wacker D, Fenalti G, Brown MA, Katritch V, Abagyan R, Cherezov V, Stevens RC (2010). Conserved binding mode of human β2 adrenergic receptor inverse agonists and antagonist revealed by X-ray crystallography. J Am Chem Soc.

[49] Rasmussen SGF, Choi H-J, Fung JJ, Pardon E, Casarosa P, Chae PS, DeVree BT, Rosenbaum DM, Thian FS, Kobilka TS, Schnapp A, Konetzki I, Sunahara RK, Gellman SH, Pautsch A, Steyaert J, Weis WI, Kobilka BK (2011). Structure of a nanobody-stabilized active state of the β(2) adrenoceptor. Nature.

[50] Rosenbaum DM, Zhang C, Lyons JA, Holl R, Aragao D, Arlow DH, Rasmussen SGF, Choi H-J, DeVree BT, Sunahara RK, Chae PS, Gellman SH, Dror RO, Shaw DE, Weis WI, Caffrey M, Gmeiner P, Kobilka BK (2011). Structure and function of an irreversible agonist-β(2) adrenoceptor complex. Nature.

[51] Zou Y, Weis WI, Kobilka BK (2012). N-terminal T4 lysozyme fusion facilitates crystallization of a G protein coupled receptor. PLoS ONE.

[52] Huang CY, Olieric V, Ma P, Howe N, Vogeley L, Liu X, Warshamanage R, Weinert T, Panepucci E, Kobilka B, Diederichs K, Wang M, Caffrey M (2016). In meso in situ serial X-ray crystallography of soluble and membrane proteins at cryogenic temperatures. Struct Biol.

[53] Ma P, Weichert D, Aleksandrov LA, Jensen TJ, Riordan JR, Liu X, Kobilka BK, Caffrey M (2017). The cubicon method for concentrating membrane proteins in the cubic mesophase. Nat Protoc.

[54] Liu X, Ahn S, Kahsai AW, Meng KC, Latorraca NR, Pani B, Venkatakrishnan AJ, Masoudi A, Weis WI, Dror RO, Chen X, Lefkowitz RJ, Kobilka BK (2017). Mechanism of intracellular allosteric β2AR antagonist revealed by X-ray crystal structure. Nature.

[55] Wang S, Che T, Levit A, Shoichet BK, Wacker D, Roth BL (2018). Structure of the D2 dopamine receptor bound to the atypical antipsychotic drug risperidone. Nature.

[56] Chien EY, Liu W, Zhao Q, Katritch V, Han GW, Hanson MA, Shi L, Newman AH, Javitch JA, Cherezov V, Stevens RC (2010). Structure of the human dopamine D3 receptor in complex with a D2/D3 selective antagonist. Science.

[57] Wang S, Wacker D, Levit A, Che T, Betz RM, McCorvy JD, Venkatakrishnan AJ, Huang XP, Dror RO, Shoichet BK, Roth BL (2017). D4 dopamine receptor high-resolution structures enable the discovery of selective agonists. Science.

[58] Shimamura T, Shiroishi M, Weyand S, Tsujimoto H, Winter G, Katritch V, Abagyan R, Cherezov V, Liu W, Han GW, Kobayashi T, Stevens RC, Iwata S (2011). Structure of the human histamine H1 receptor complex with doxepin. Nature.

[59] LigPrep, Schrodinger Release 2018-1, LLC, New York, NY, 2018

[60] Moriwaki H, Tian Y-S, Kawashita N, Takagi T (2018). Mordred: a molecular descriptor calculator. J Cheminf.

