# Supplementary figures and images for: Three-dimensional descriptors for aminergic GPCRs: dependence on docking conformation and crystal structure
Source: Mol Divers. 2018 Nov 27;23(3):603–13. doi: 10.1007/s11030-018-9894-4 (PMC6682580; doi:10.1007/s11030-018-9894-4)

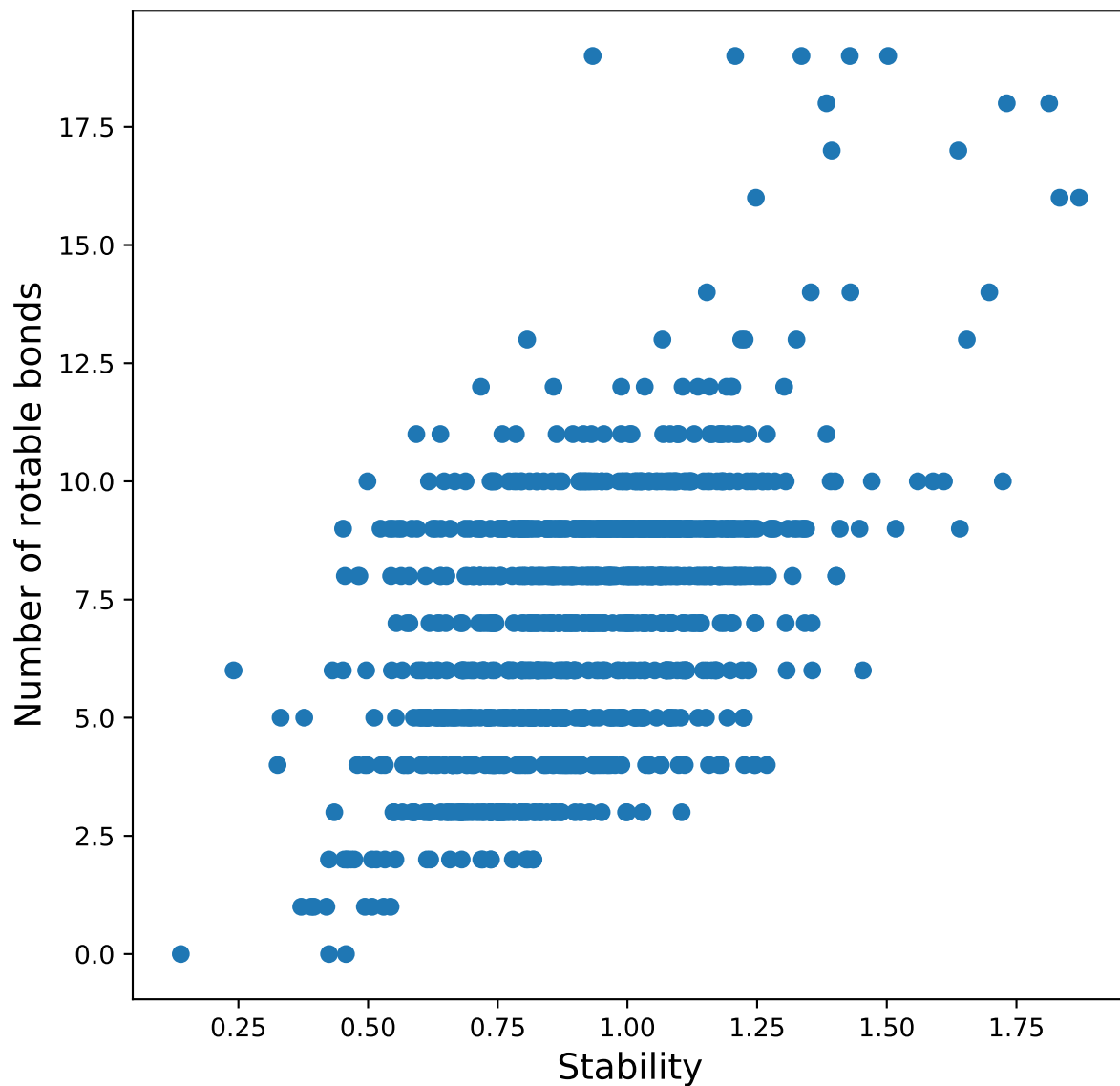

Supplement: Supplementary file 2 — Visualization of the correlation between the number of rotatable bonds and the variations in atom positions in the docked poses (ZIP 1316 kb) [file 11030_2018_9894_MOESM2_ESM.zip › Supplementary_File_S2/stability_pose_K=10_5HT1B.pdf]

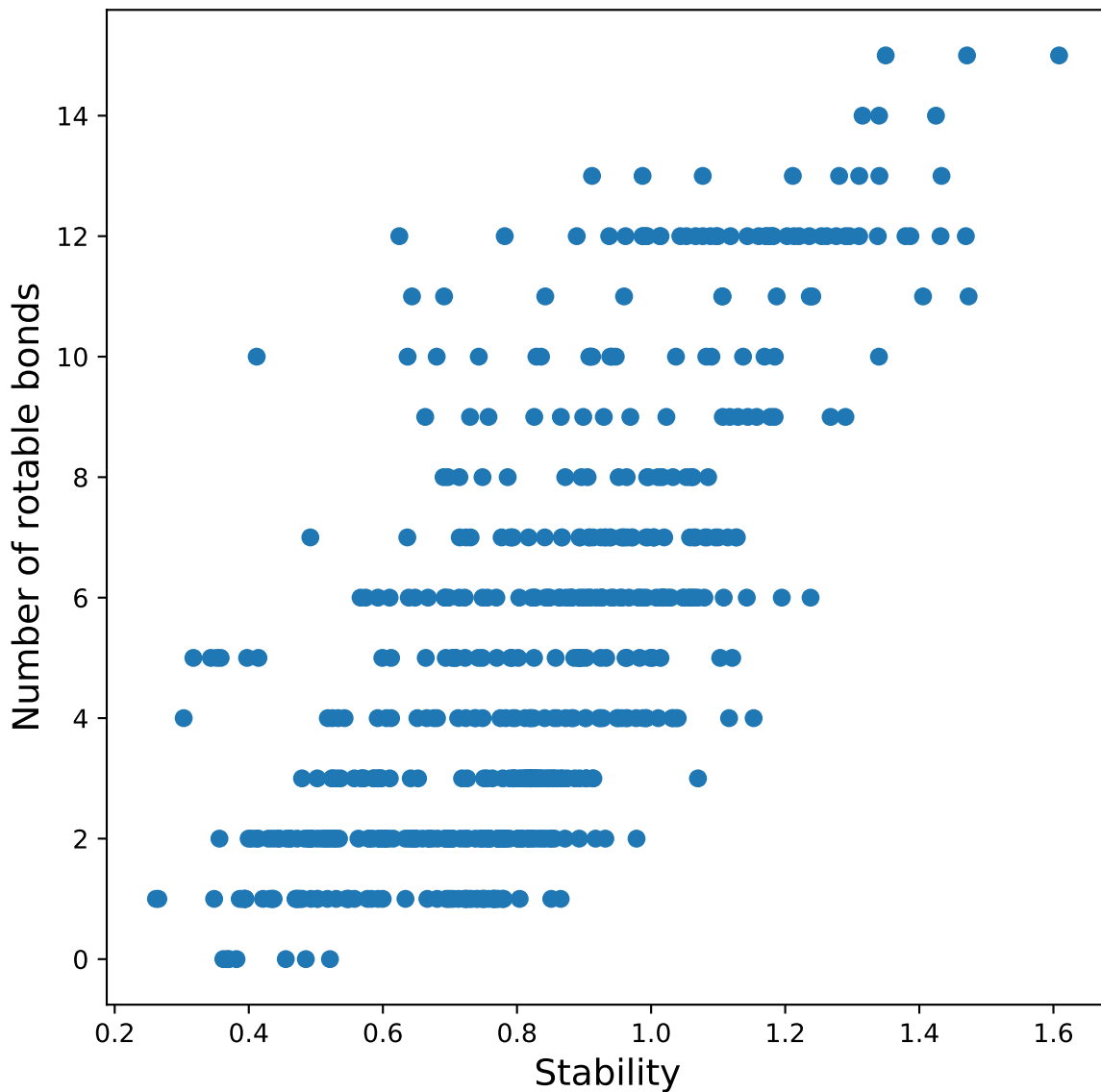

Supplement: Supplementary file 2 — Visualization of the correlation between the number of rotatable bonds and the variations in atom positions in the docked poses (ZIP 1316 kb) [file 11030_2018_9894_MOESM2_ESM.zip › Supplementary_File_S2/stability_pose_K=10_5HT2B.pdf]

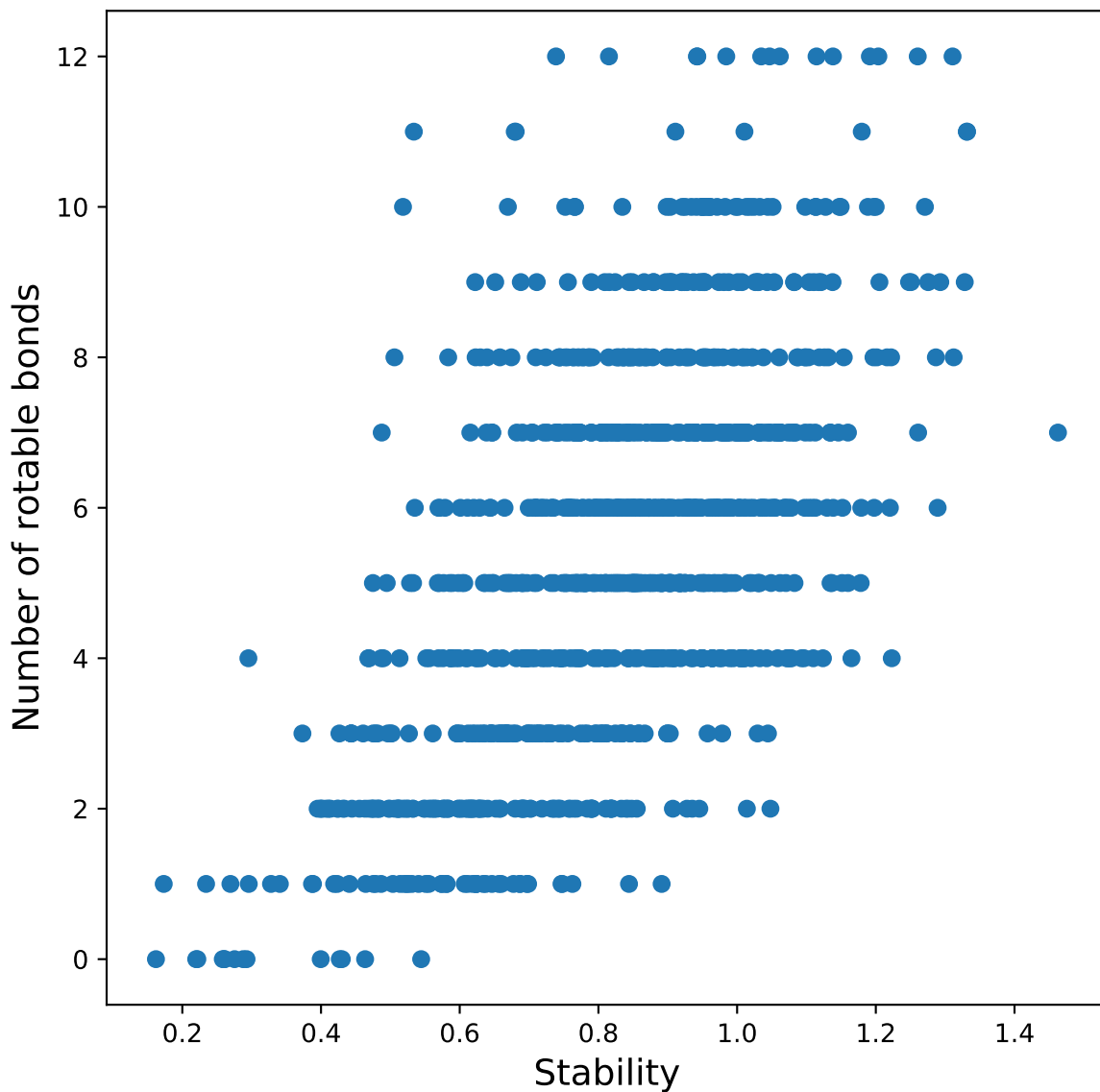

Supplement: Supplementary file 2 — Visualization of the correlation between the number of rotatable bonds and the variations in atom positions in the docked poses (ZIP 1316 kb) [file 11030_2018_9894_MOESM2_ESM.zip › Supplementary_File_S2/stability_pose_K=10_5HT2C.pdf]

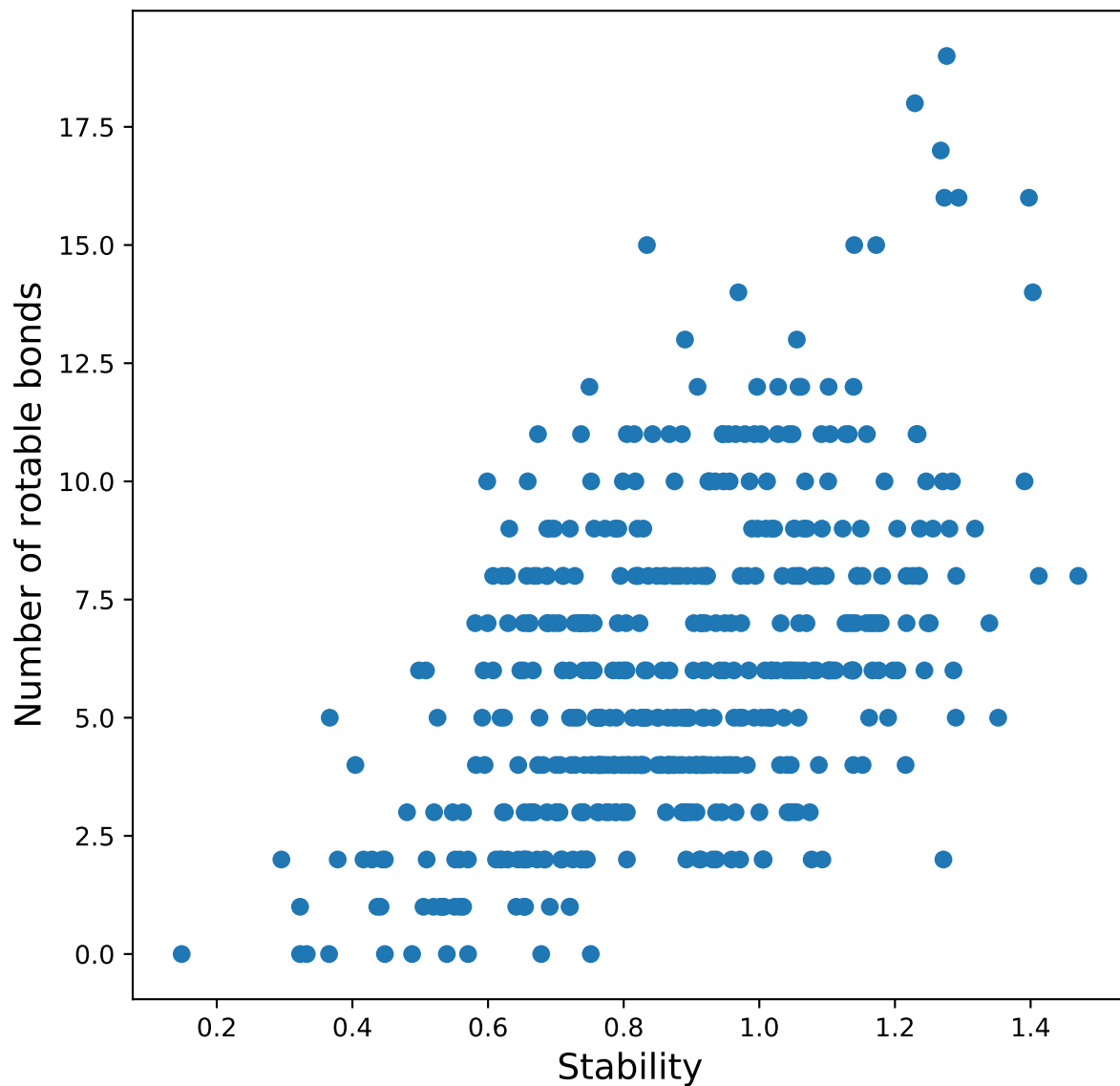

Supplement: Supplementary file 2 — Visualization of the correlation between the number of rotatable bonds and the variations in atom positions in the docked poses (ZIP 1316 kb) [file 11030_2018_9894_MOESM2_ESM.zip › Supplementary_File_S2/stability_pose_K=10_ACM1.pdf]

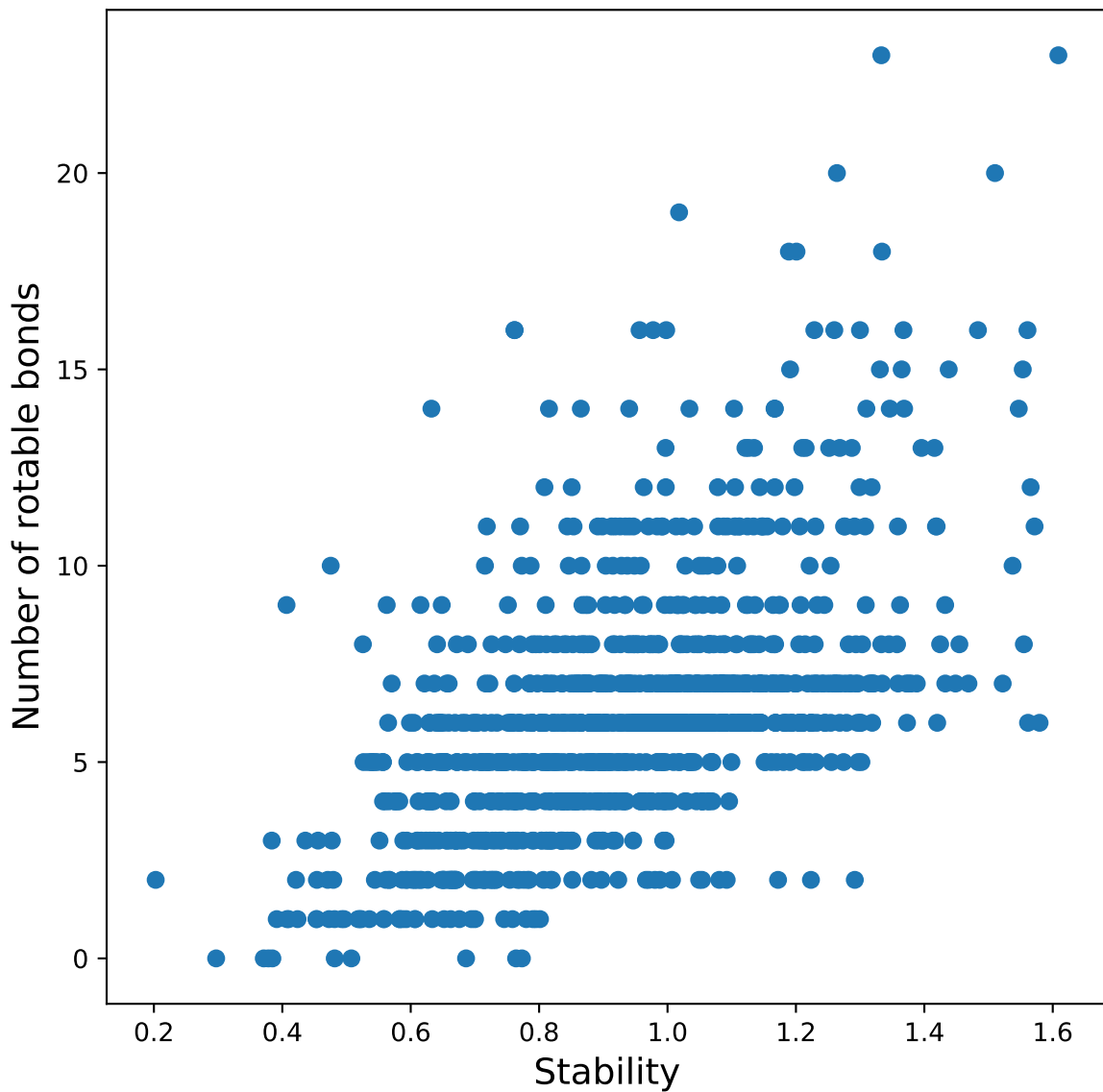

Supplement: Supplementary file 2 — Visualization of the correlation between the number of rotatable bonds and the variations in atom positions in the docked poses (ZIP 1316 kb) [file 11030_2018_9894_MOESM2_ESM.zip › Supplementary_File_S2/stability_pose_K=10_ACM2.pdf]

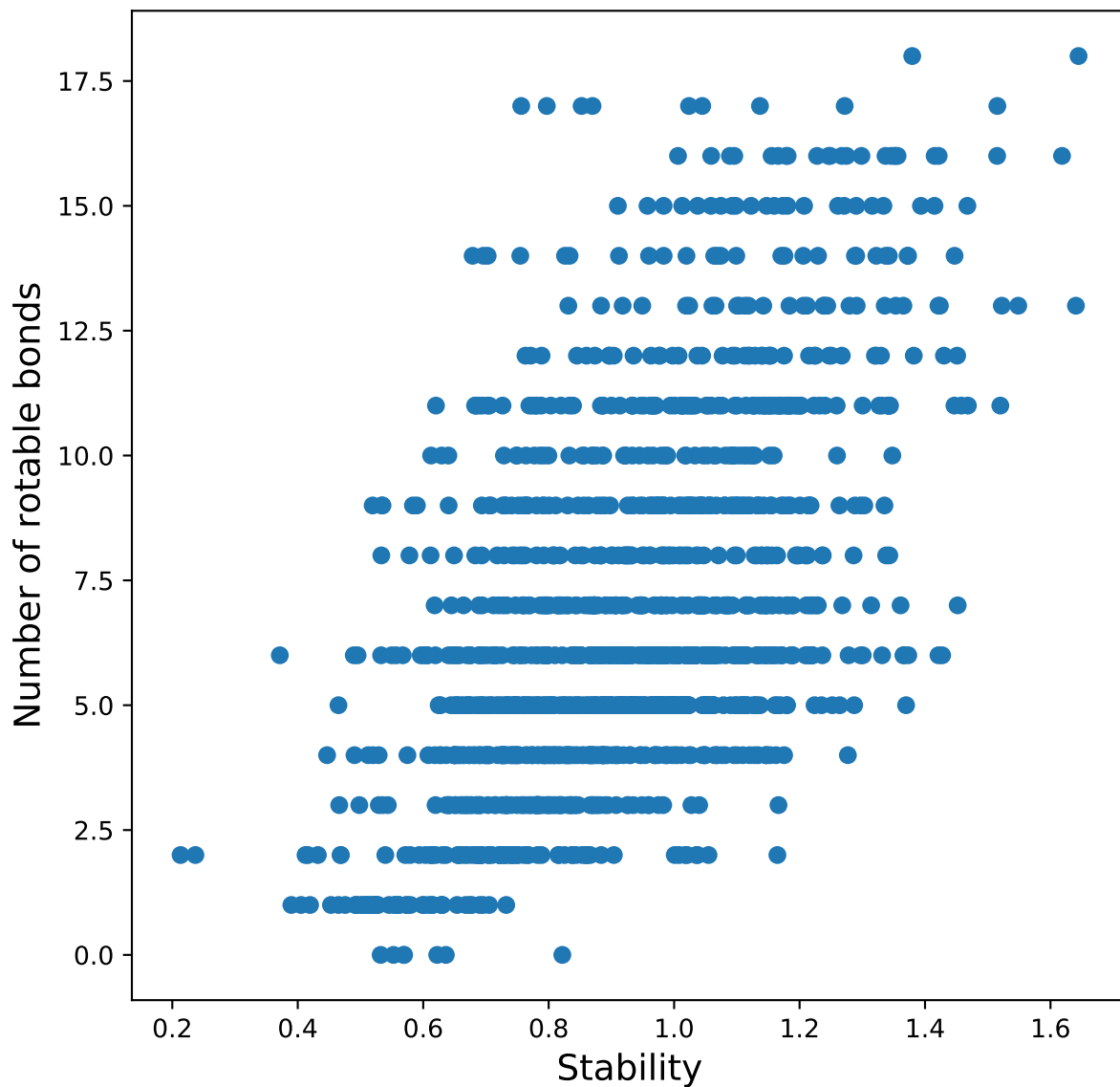

Supplement: Supplementary file 2 — Visualization of the correlation between the number of rotatable bonds and the variations in atom positions in the docked poses (ZIP 1316 kb) [file 11030_2018_9894_MOESM2_ESM.zip › Supplementary_File_S2/stability_pose_K=10_ACM3.pdf]

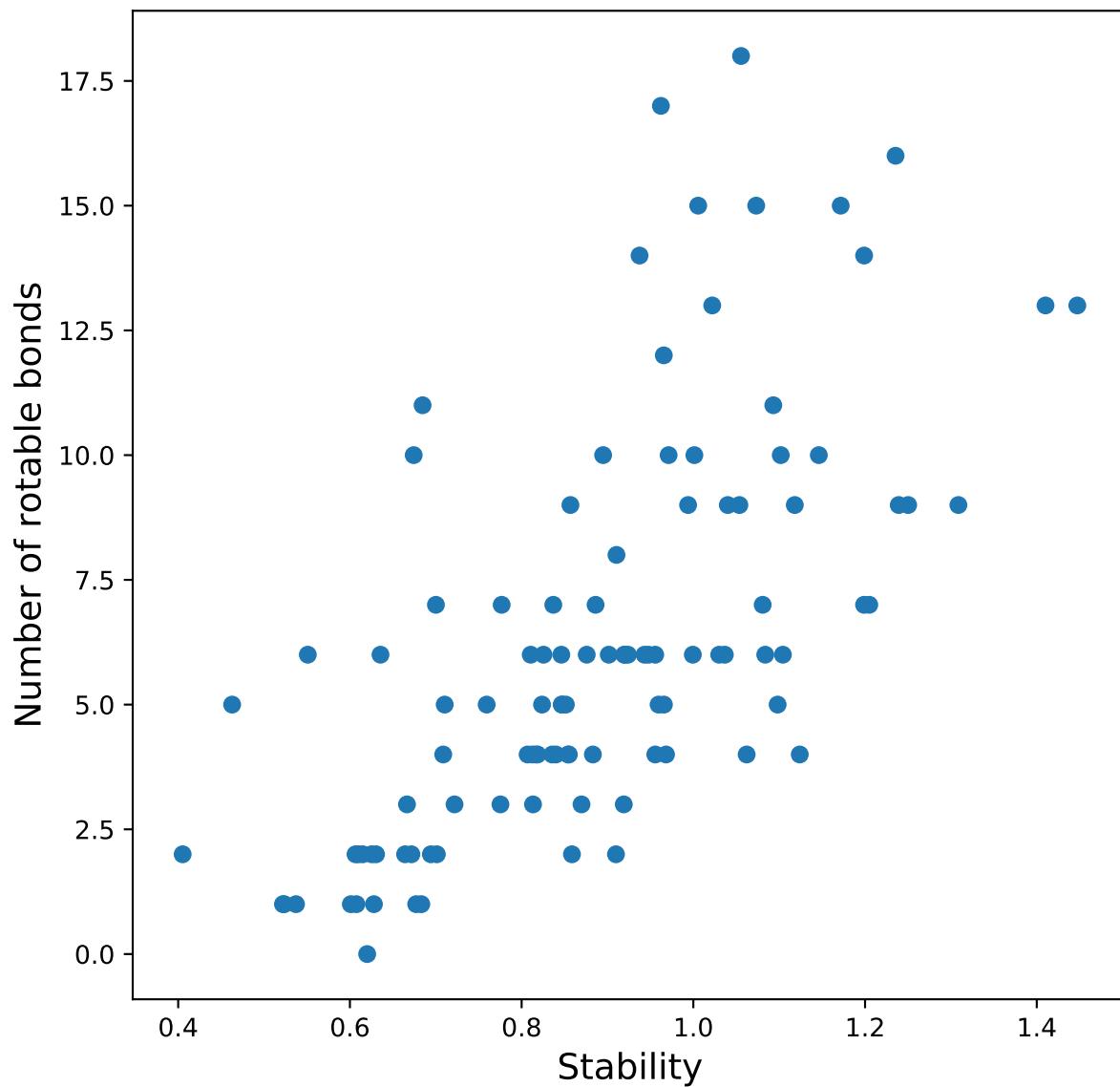

Supplement: Supplementary file 2 — Visualization of the correlation between the number of rotatable bonds and the variations in atom positions in the docked poses (ZIP 1316 kb) [file 11030_2018_9894_MOESM2_ESM.zip › Supplementary_File_S2/stability_pose_K=10_ACM4.pdf]

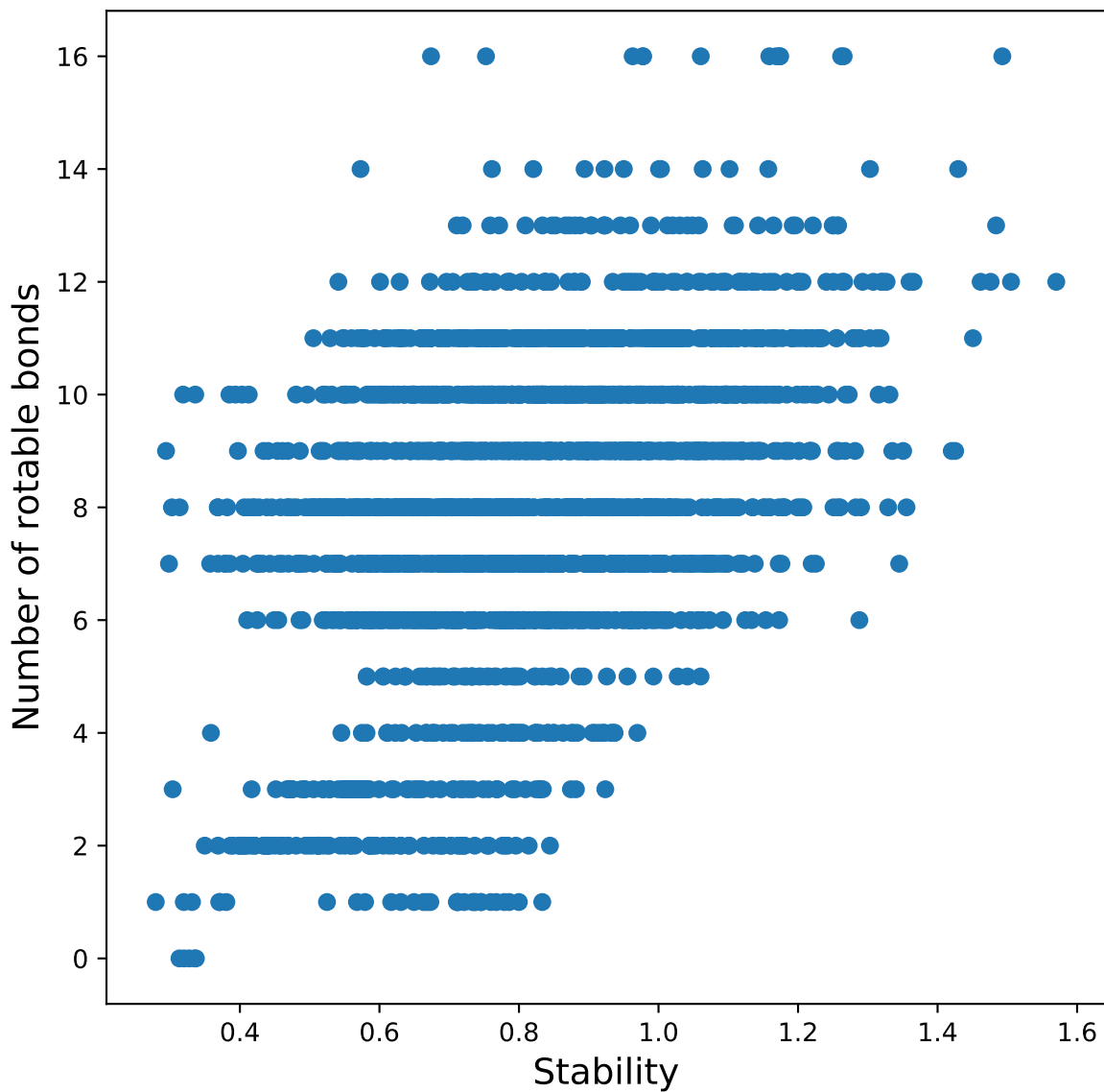

Supplement: Supplementary file 2 — Visualization of the correlation between the number of rotatable bonds and the variations in atom positions in the docked poses (ZIP 1316 kb) [file 11030_2018_9894_MOESM2_ESM.zip › Supplementary_File_S2/stability_pose_K=10_BETA1.pdf]

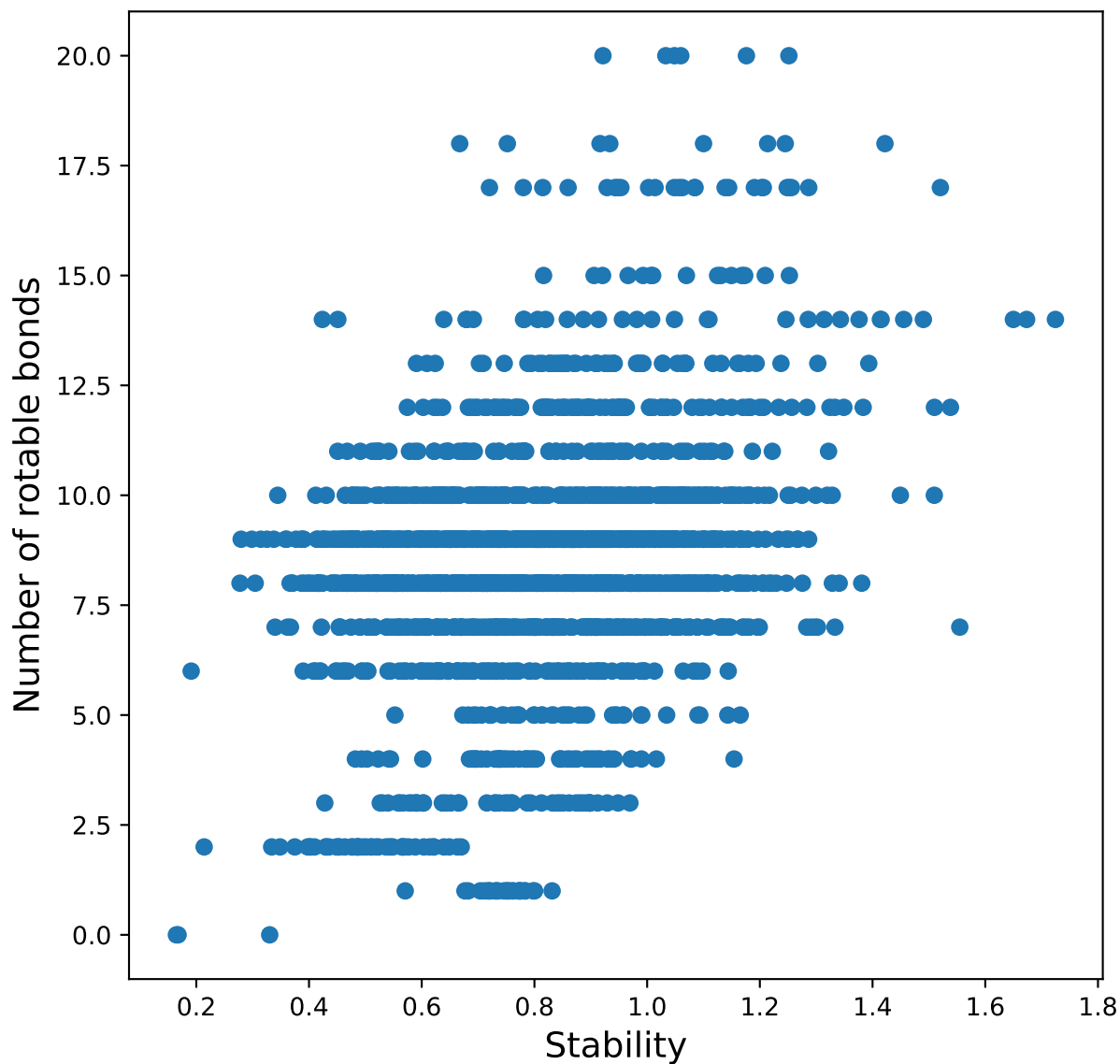

Supplement: Supplementary file 2 — Visualization of the correlation between the number of rotatable bonds and the variations in atom positions in the docked poses (ZIP 1316 kb) [file 11030_2018_9894_MOESM2_ESM.zip › Supplementary_File_S2/stability_pose_K=10_BETA2.pdf]

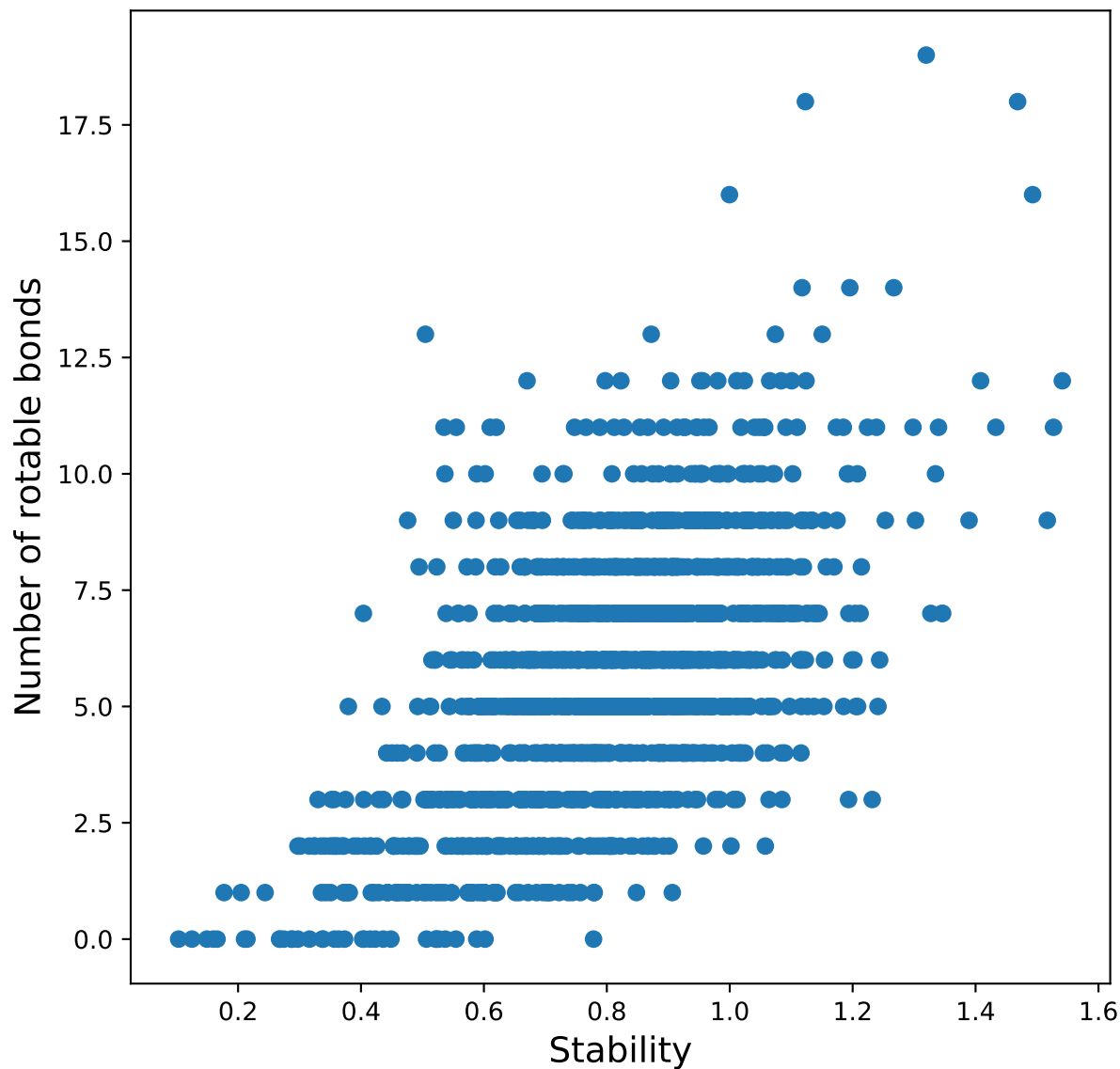

Supplement: Supplementary file 2 — Visualization of the correlation between the number of rotatable bonds and the variations in atom positions in the docked poses (ZIP 1316 kb) [file 11030_2018_9894_MOESM2_ESM.zip › Supplementary_File_S2/stability_pose_K=10_D2.pdf]

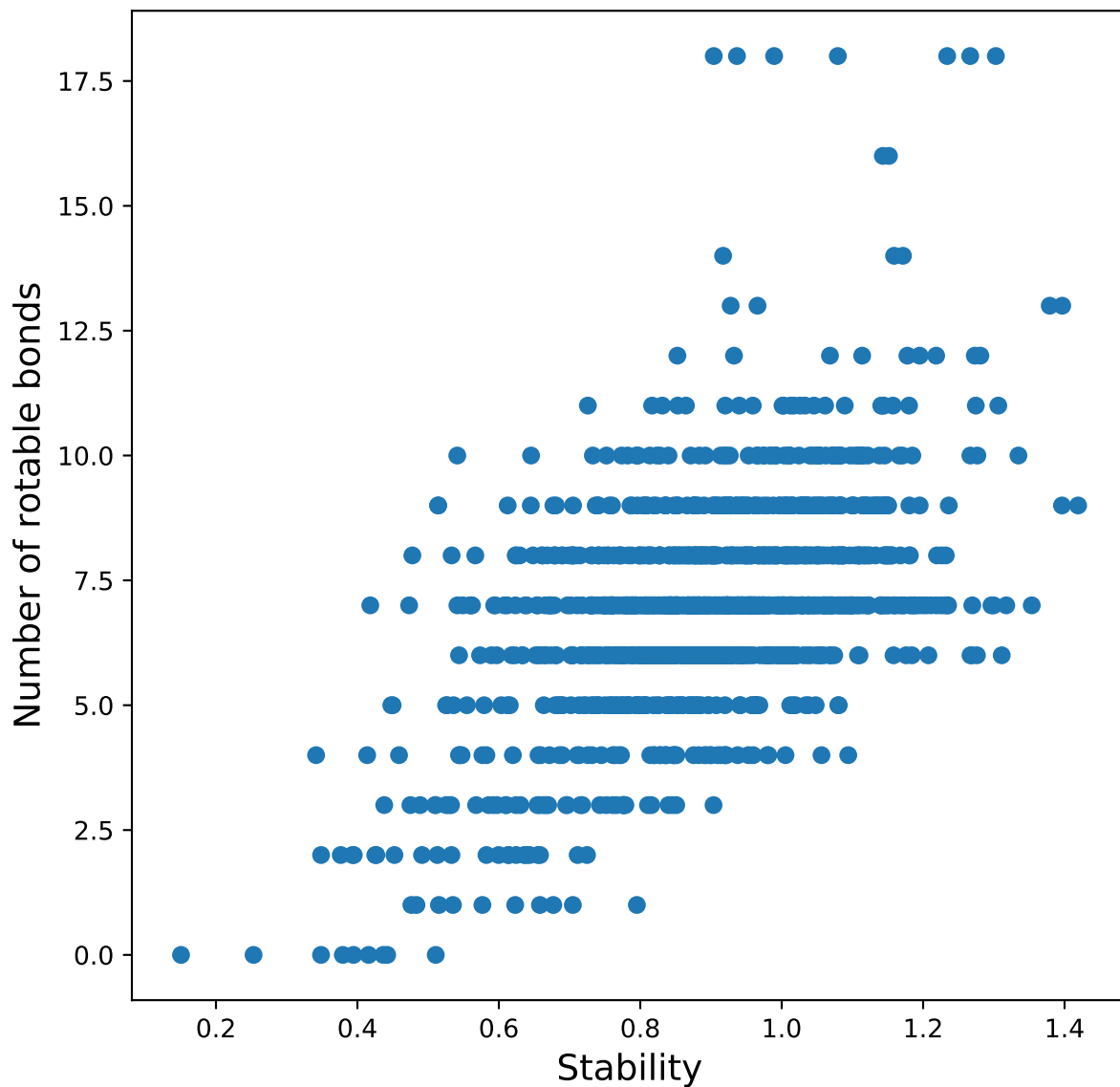

Supplement: Supplementary file 2 — Visualization of the correlation between the number of rotatable bonds and the variations in atom positions in the docked poses (ZIP 1316 kb) [file 11030_2018_9894_MOESM2_ESM.zip › Supplementary_File_S2/stability_pose_K=10_D3.pdf]

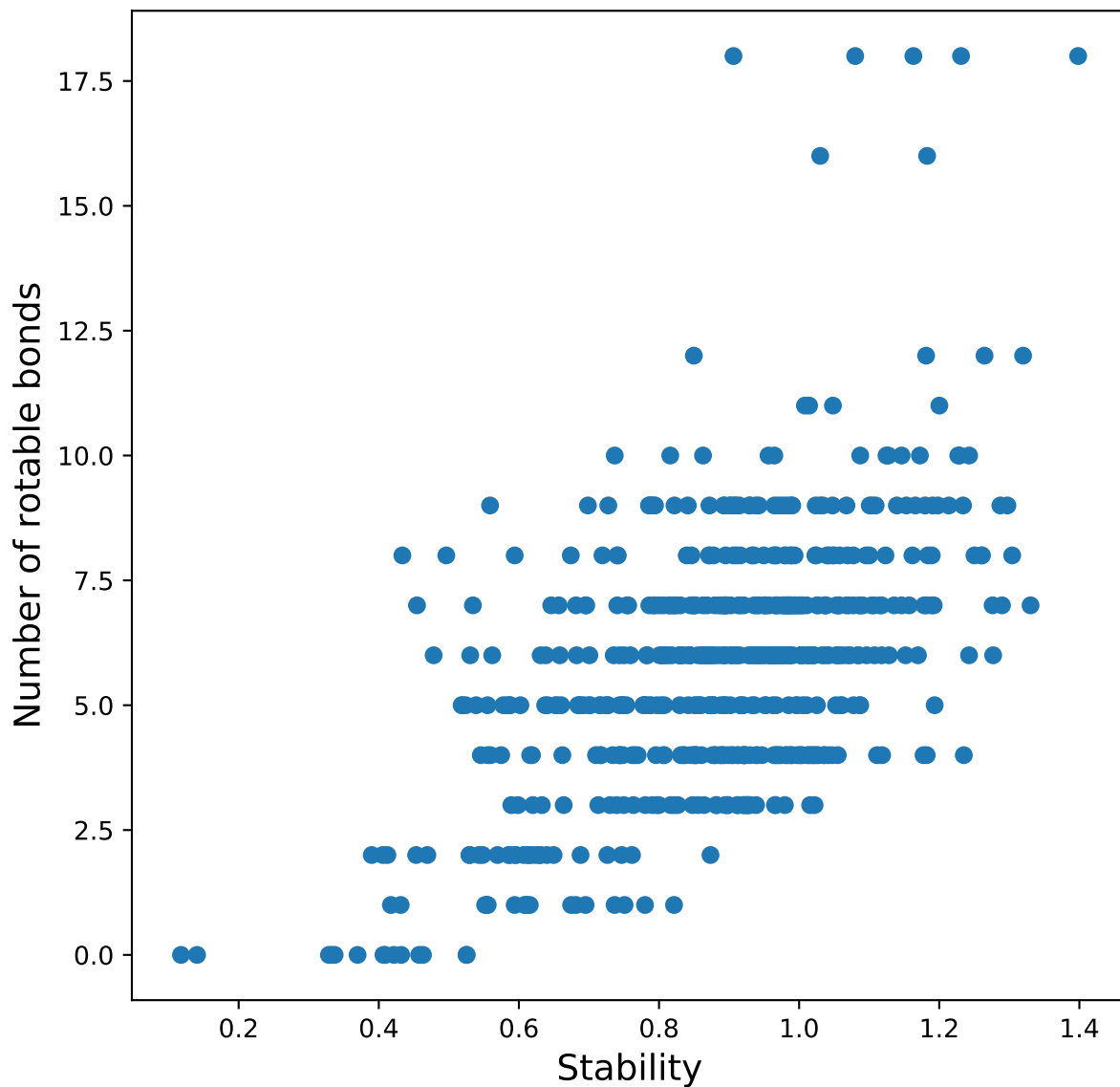

Supplement: Supplementary file 2 — Visualization of the correlation between the number of rotatable bonds and the variations in atom positions in the docked poses (ZIP 1316 kb) [file 11030_2018_9894_MOESM2_ESM.zip › Supplementary_File_S2/stability_pose_K=10_D4.pdf]

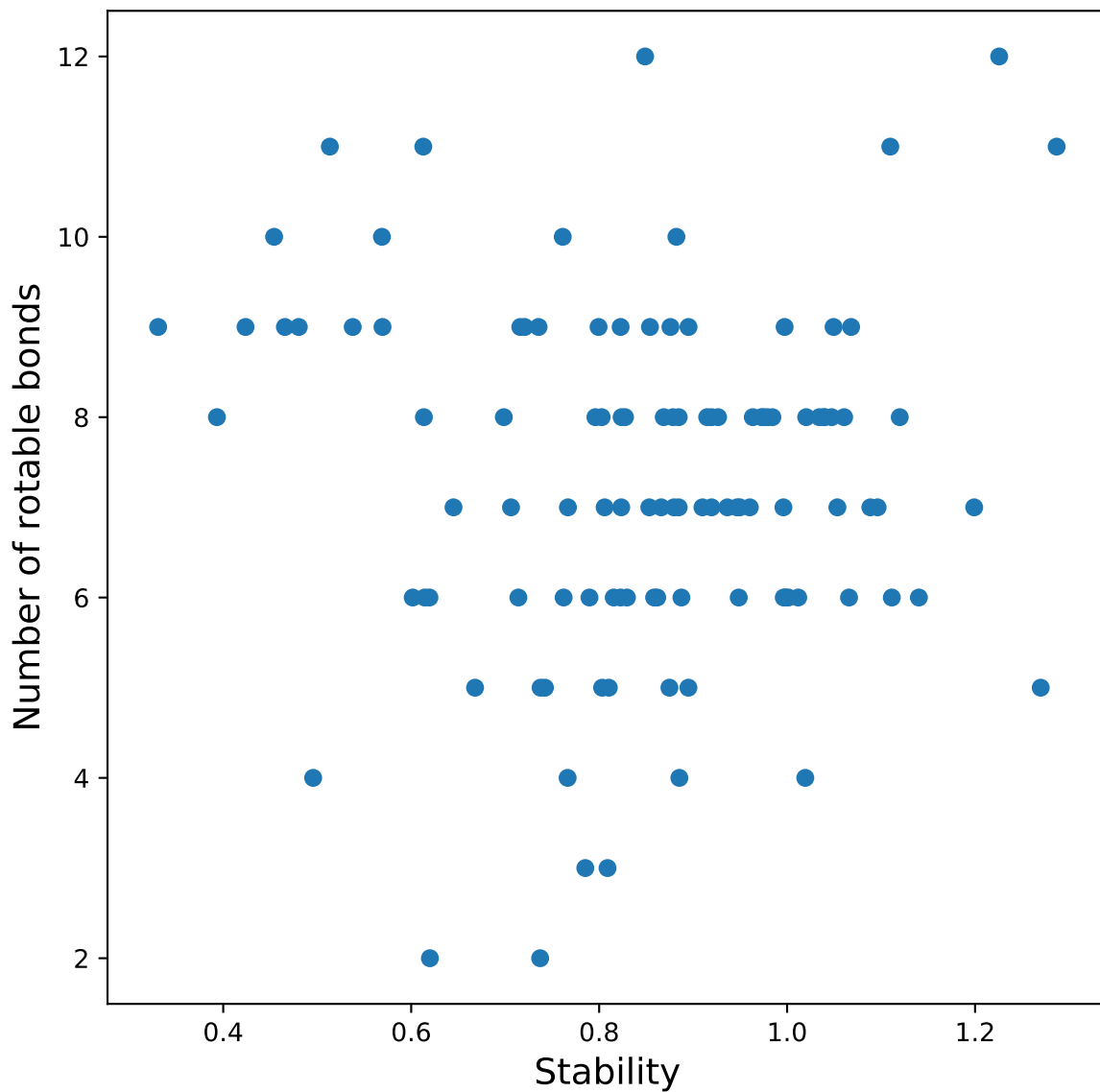

Supplement: Supplementary file 2 — Visualization of the correlation between the number of rotatable bonds and the variations in atom positions in the docked poses (ZIP 1316 kb) [file 11030_2018_9894_MOESM2_ESM.zip › Supplementary_File_S2/stability_pose_K=10_H1.pdf]

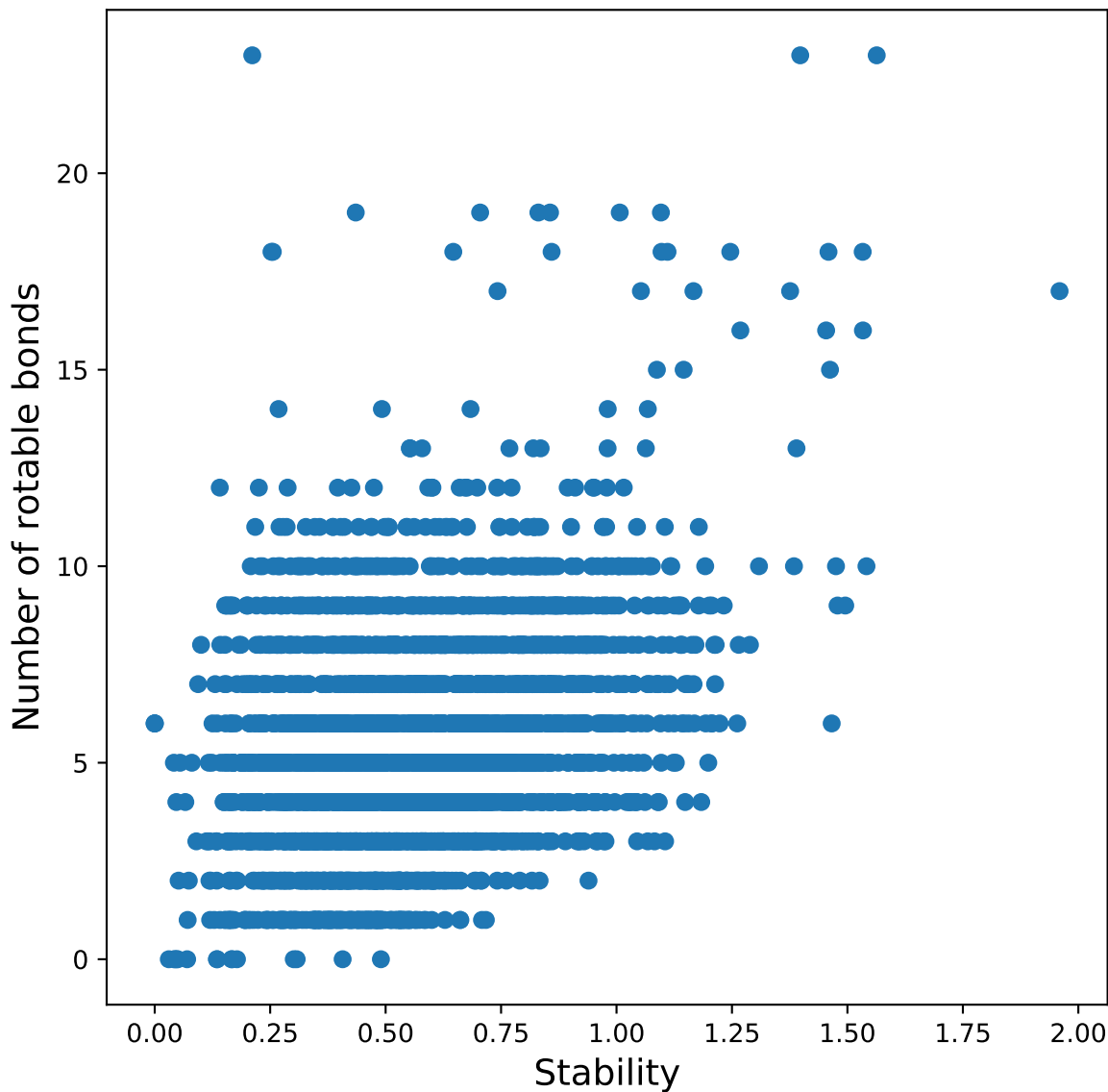

Supplement: Supplementary file 2 — Visualization of the correlation between the number of rotatable bonds and the variations in atom positions in the docked poses (ZIP 1316 kb) [file 11030_2018_9894_MOESM2_ESM.zip › Supplementary_File_S2/stability_pose_K=3_5HT1B.pdf]

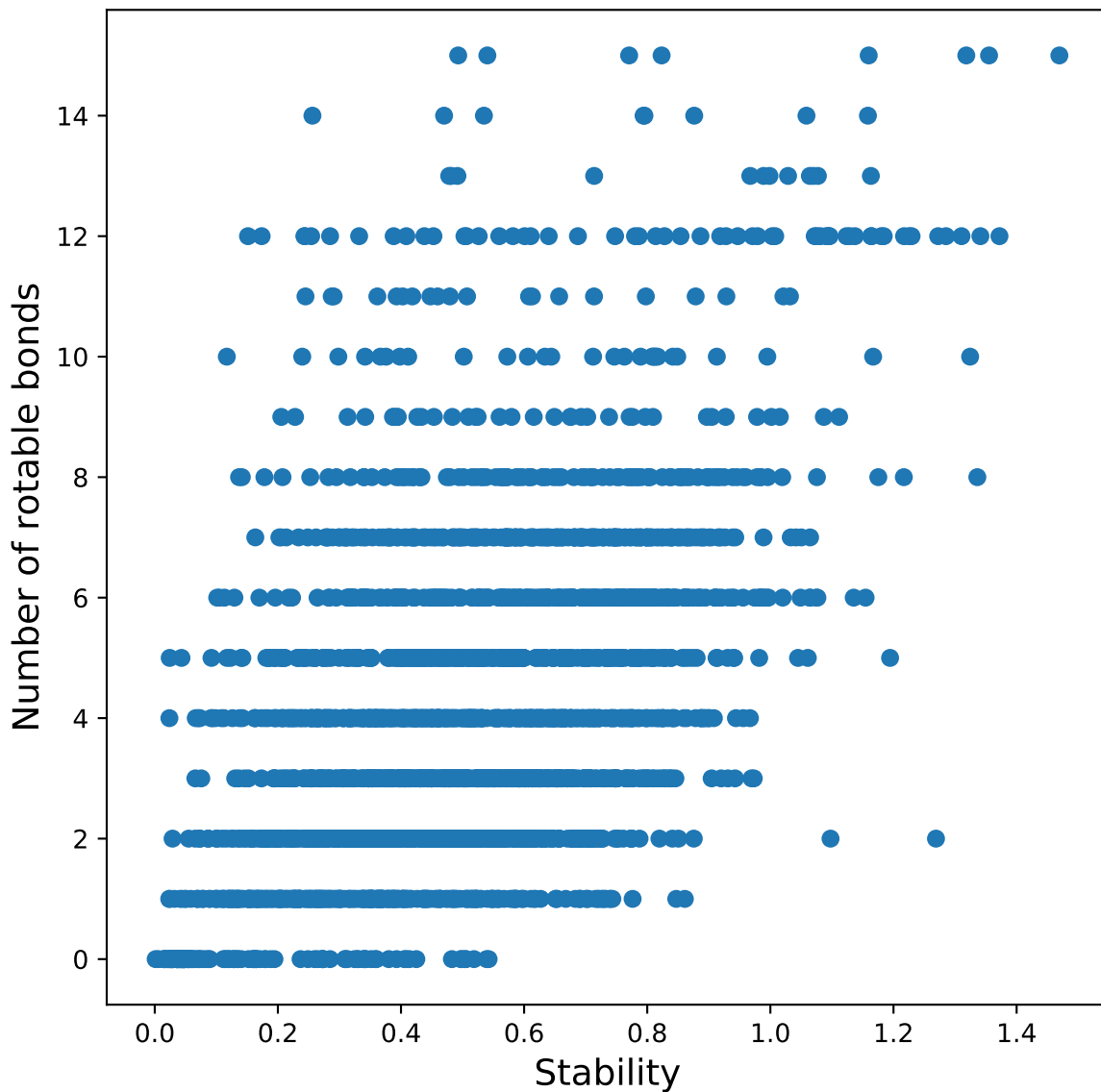

Supplement: Supplementary file 2 — Visualization of the correlation between the number of rotatable bonds and the variations in atom positions in the docked poses (ZIP 1316 kb) [file 11030_2018_9894_MOESM2_ESM.zip › Supplementary_File_S2/stability_pose_K=3_5HT2B.pdf]

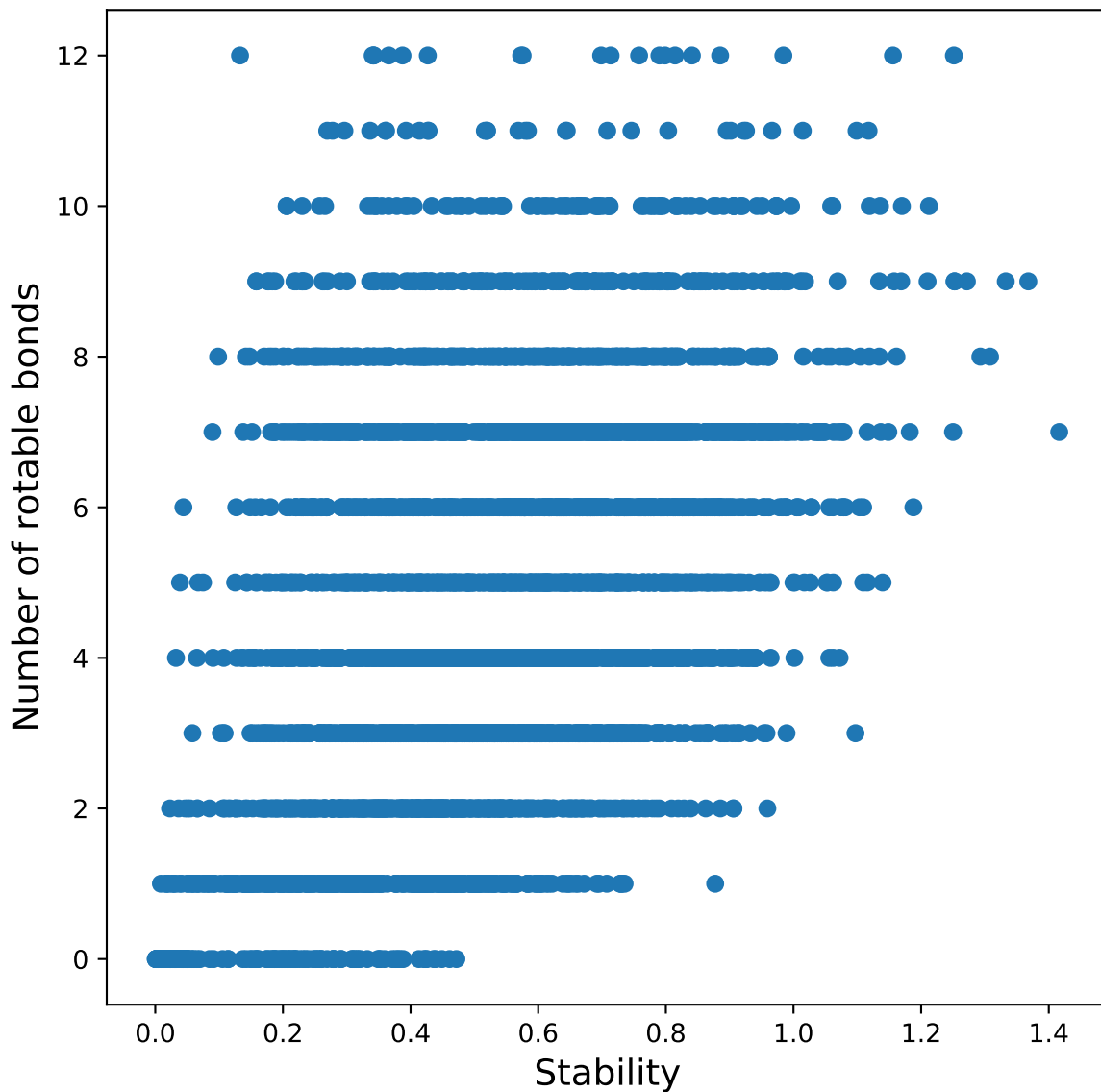

Supplement: Supplementary file 2 — Visualization of the correlation between the number of rotatable bonds and the variations in atom positions in the docked poses (ZIP 1316 kb) [file 11030_2018_9894_MOESM2_ESM.zip › Supplementary_File_S2/stability_pose_K=3_5HT2C.pdf]

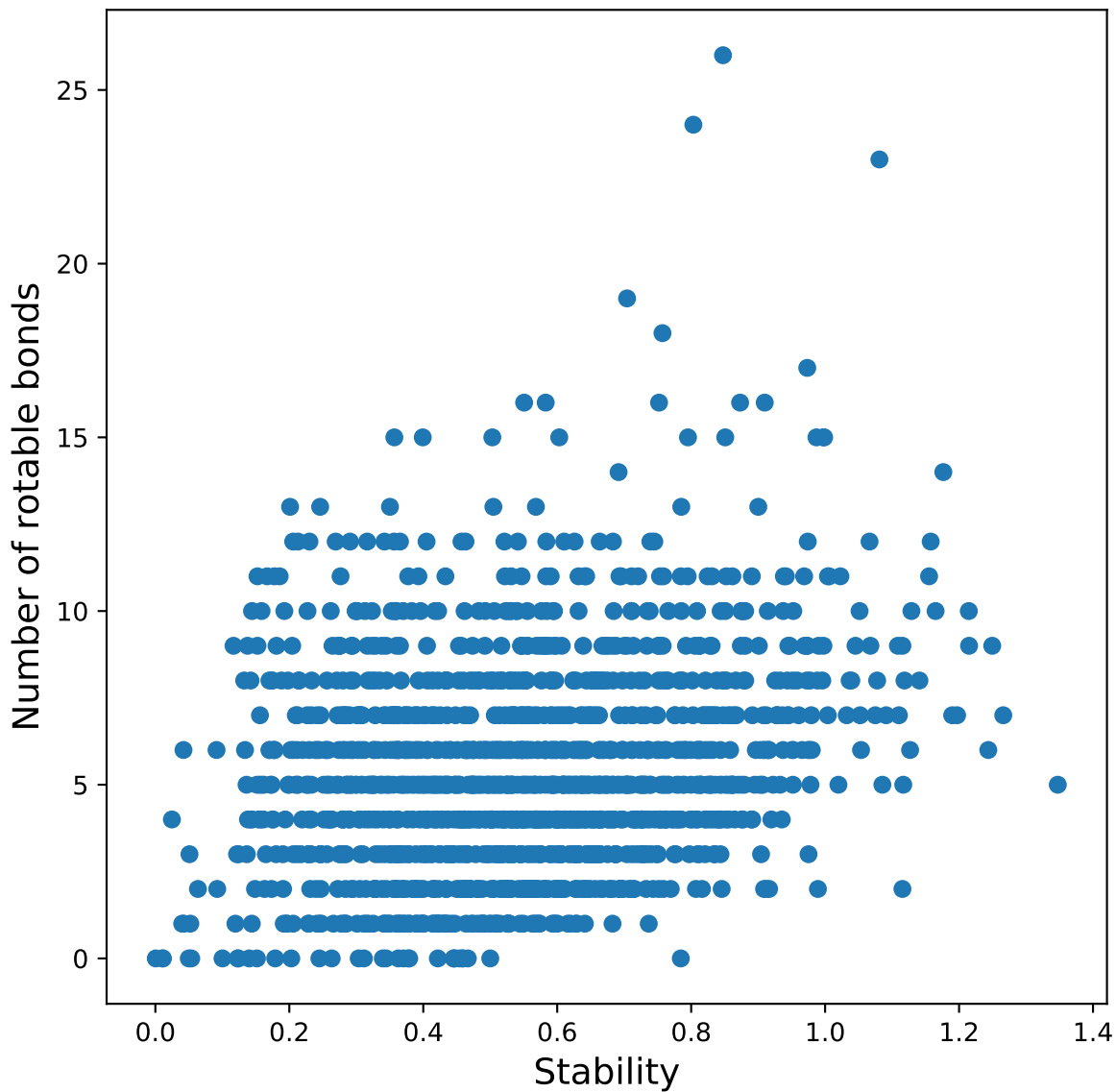

Supplement: Supplementary file 2 — Visualization of the correlation between the number of rotatable bonds and the variations in atom positions in the docked poses (ZIP 1316 kb) [file 11030_2018_9894_MOESM2_ESM.zip › Supplementary_File_S2/stability_pose_K=3_ACM1.pdf]

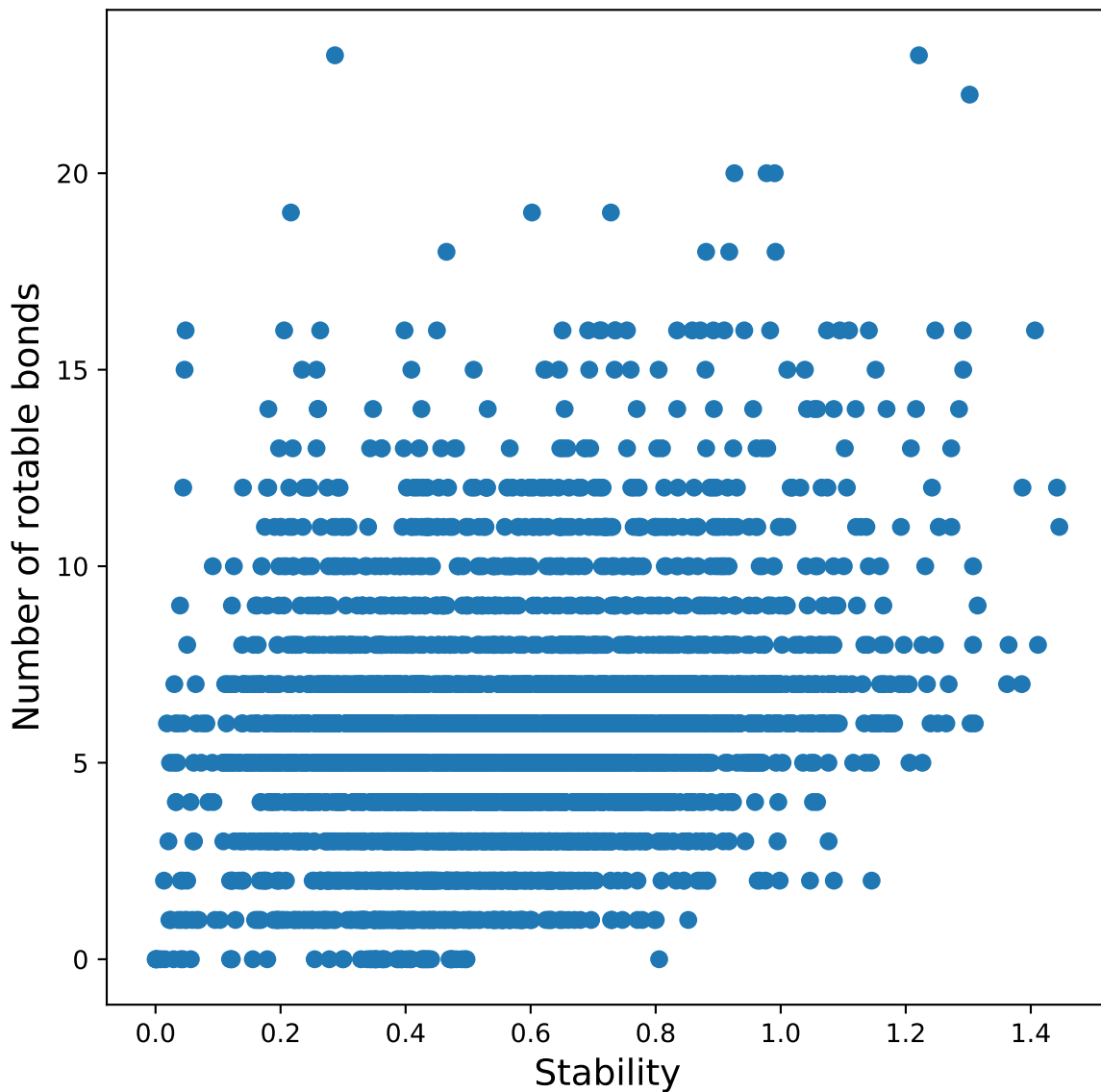

Supplement: Supplementary file 2 — Visualization of the correlation between the number of rotatable bonds and the variations in atom positions in the docked poses (ZIP 1316 kb) [file 11030_2018_9894_MOESM2_ESM.zip › Supplementary_File_S2/stability_pose_K=3_ACM2.pdf]

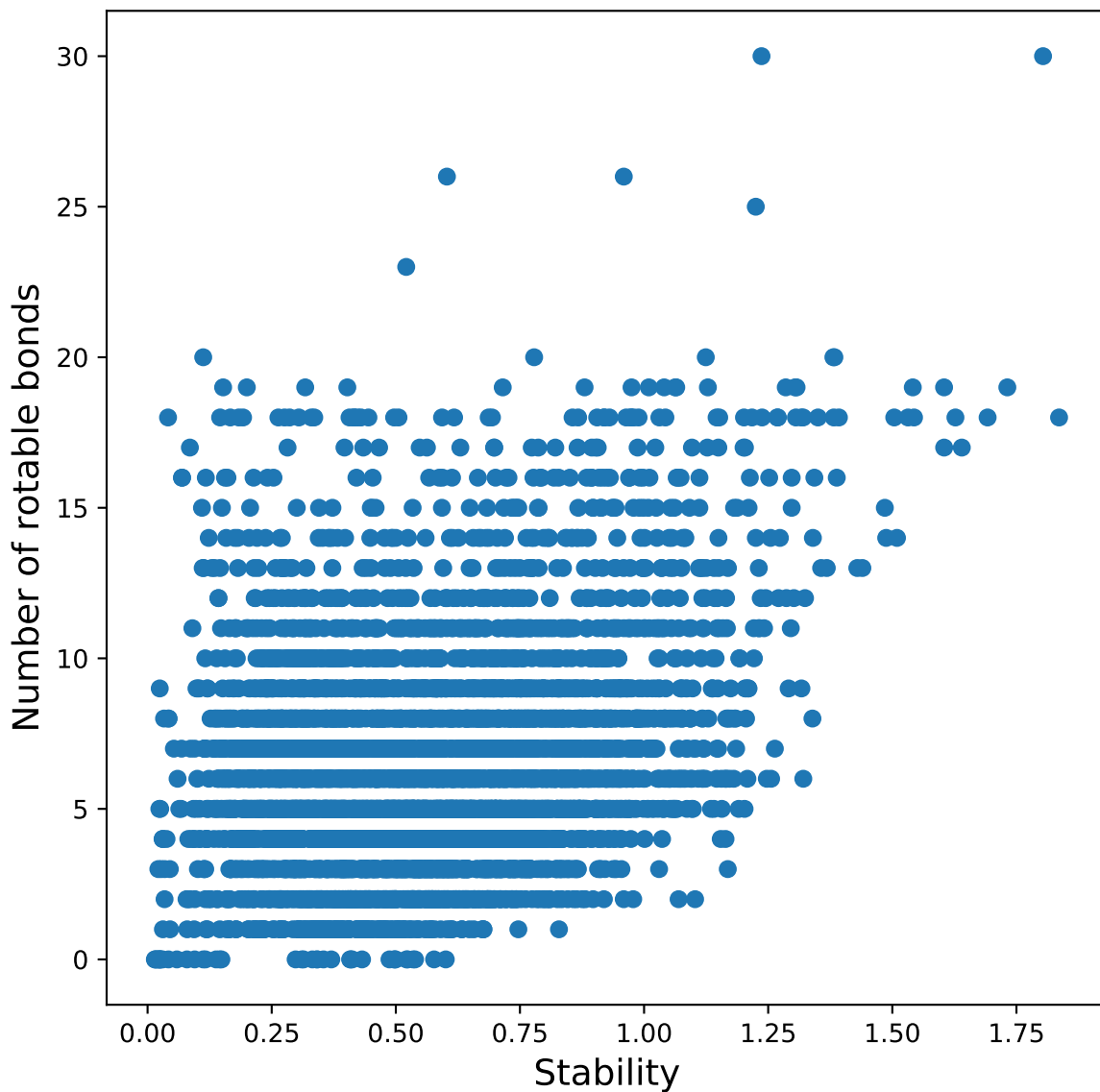

Supplement: Supplementary file 2 — Visualization of the correlation between the number of rotatable bonds and the variations in atom positions in the docked poses (ZIP 1316 kb) [file 11030_2018_9894_MOESM2_ESM.zip › Supplementary_File_S2/stability_pose_K=3_ACM3.pdf]

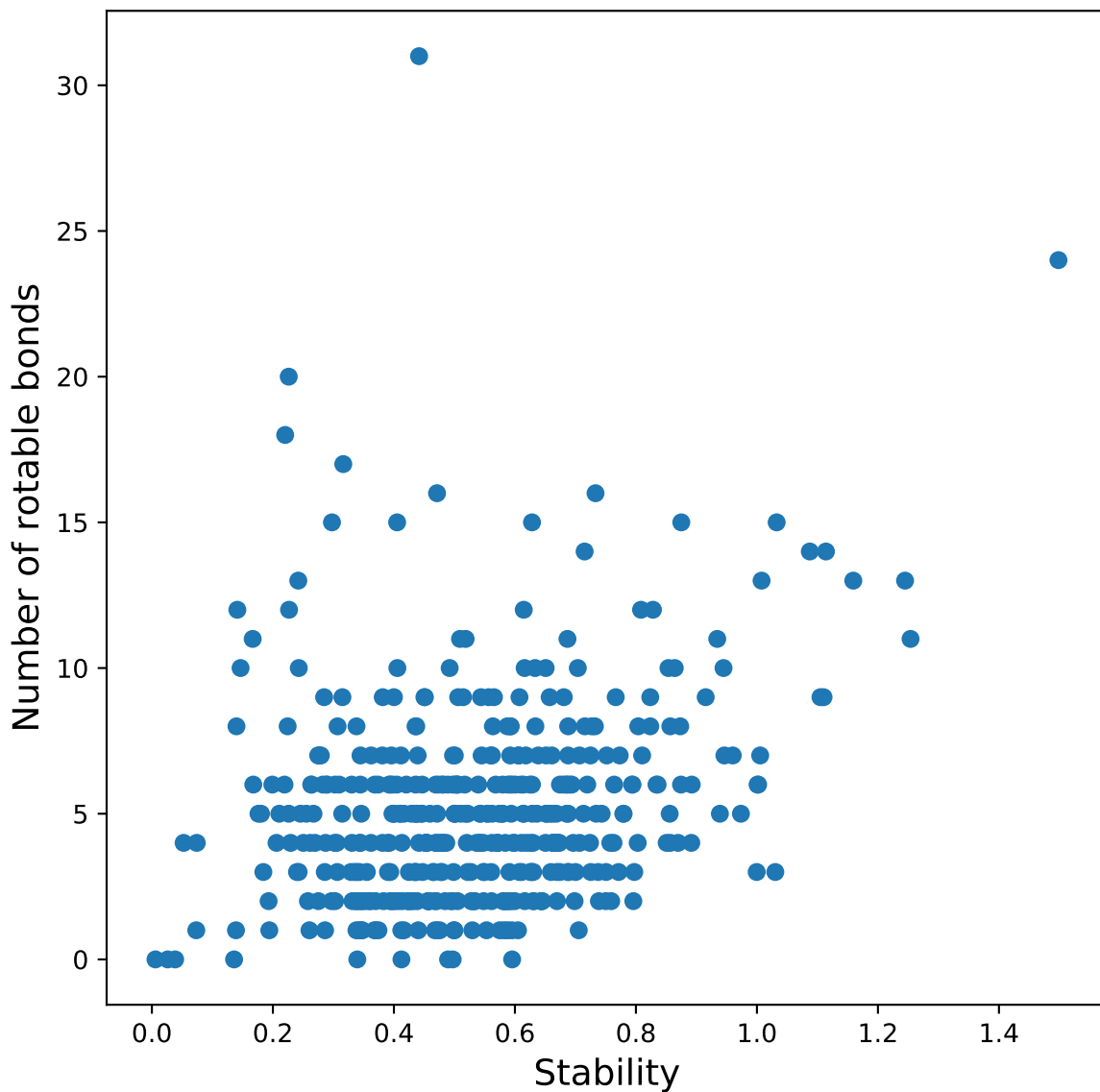

Supplement: Supplementary file 2 — Visualization of the correlation between the number of rotatable bonds and the variations in atom positions in the docked poses (ZIP 1316 kb) [file 11030_2018_9894_MOESM2_ESM.zip › Supplementary_File_S2/stability_pose_K=3_ACM4.pdf]

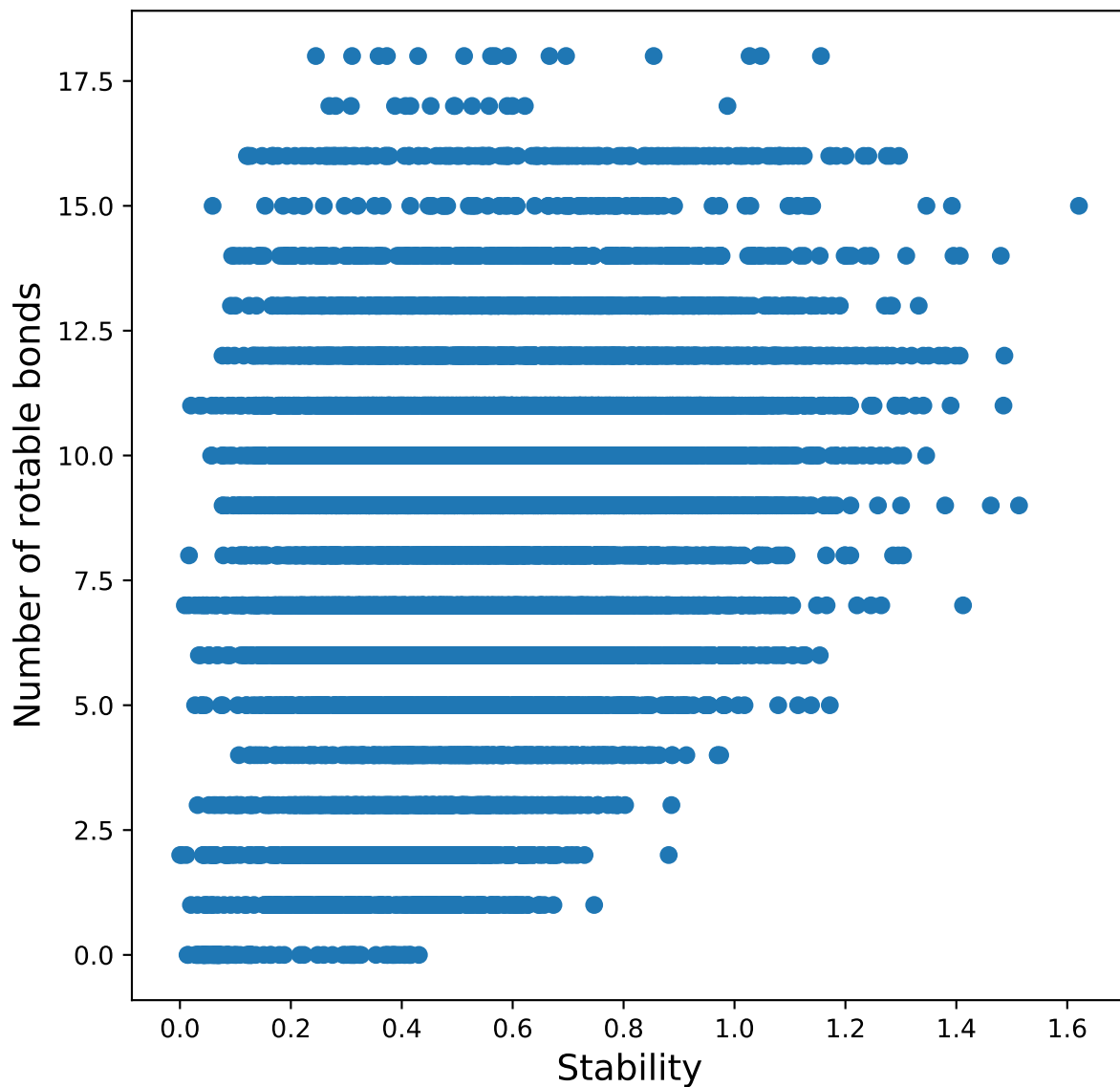

Supplement: Supplementary file 2 — Visualization of the correlation between the number of rotatable bonds and the variations in atom positions in the docked poses (ZIP 1316 kb) [file 11030_2018_9894_MOESM2_ESM.zip › Supplementary_File_S2/stability_pose_K=3_BETA1.pdf]

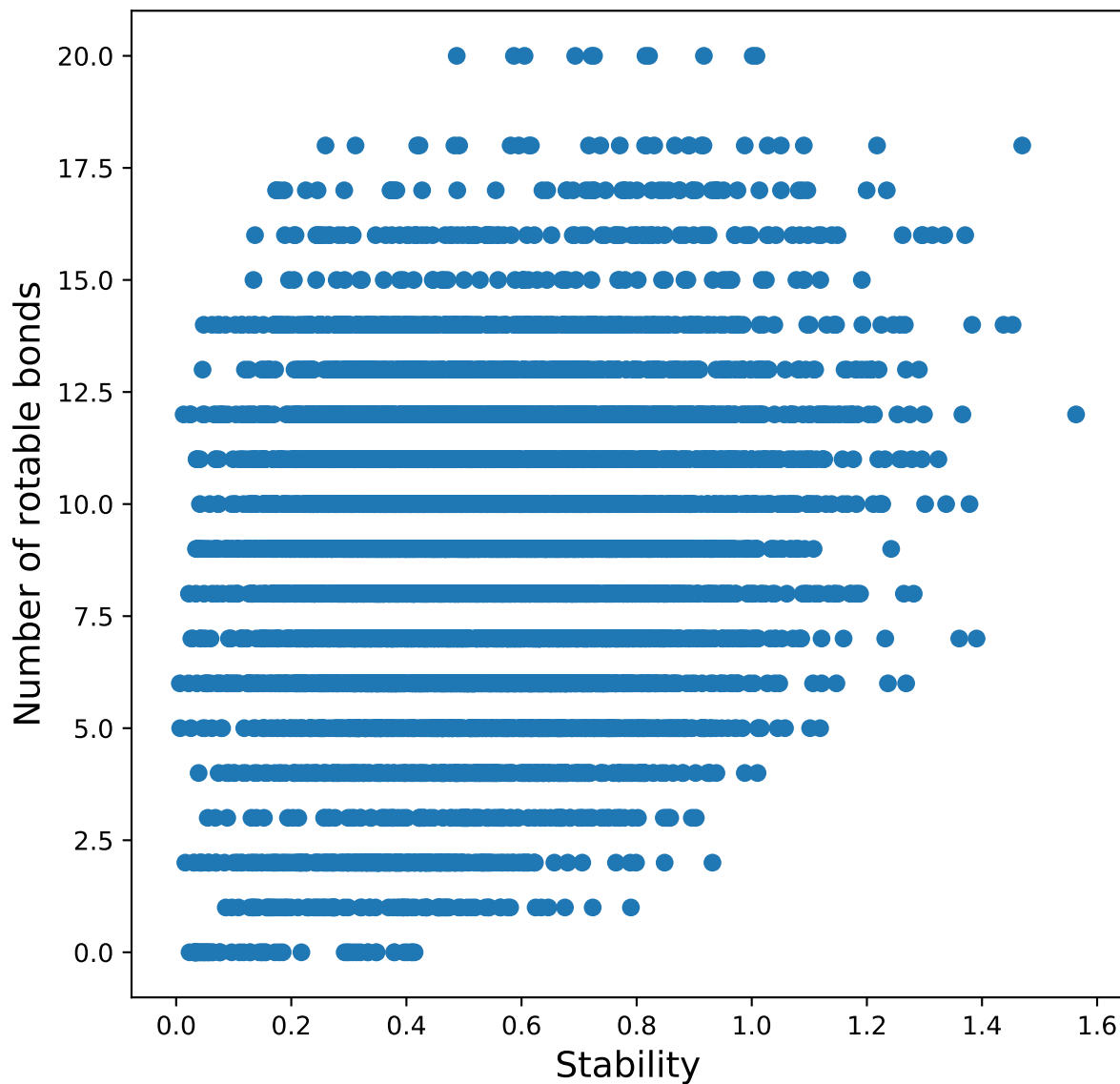

Supplement: Supplementary file 2 — Visualization of the correlation between the number of rotatable bonds and the variations in atom positions in the docked poses (ZIP 1316 kb) [file 11030_2018_9894_MOESM2_ESM.zip › Supplementary_File_S2/stability_pose_K=3_BETA2.pdf]

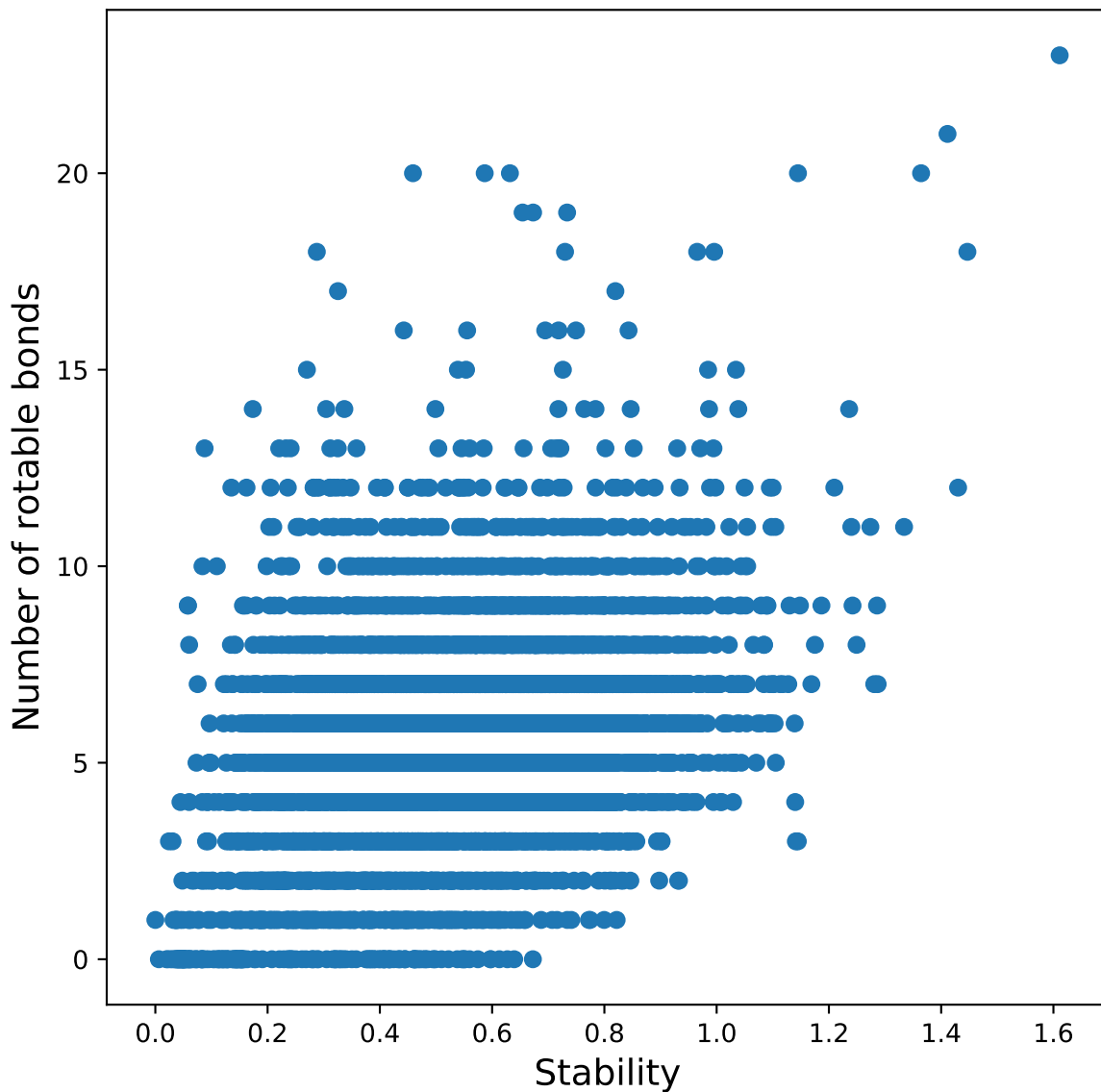

Supplement: Supplementary file 2 — Visualization of the correlation between the number of rotatable bonds and the variations in atom positions in the docked poses (ZIP 1316 kb) [file 11030_2018_9894_MOESM2_ESM.zip › Supplementary_File_S2/stability_pose_K=3_D2.pdf]

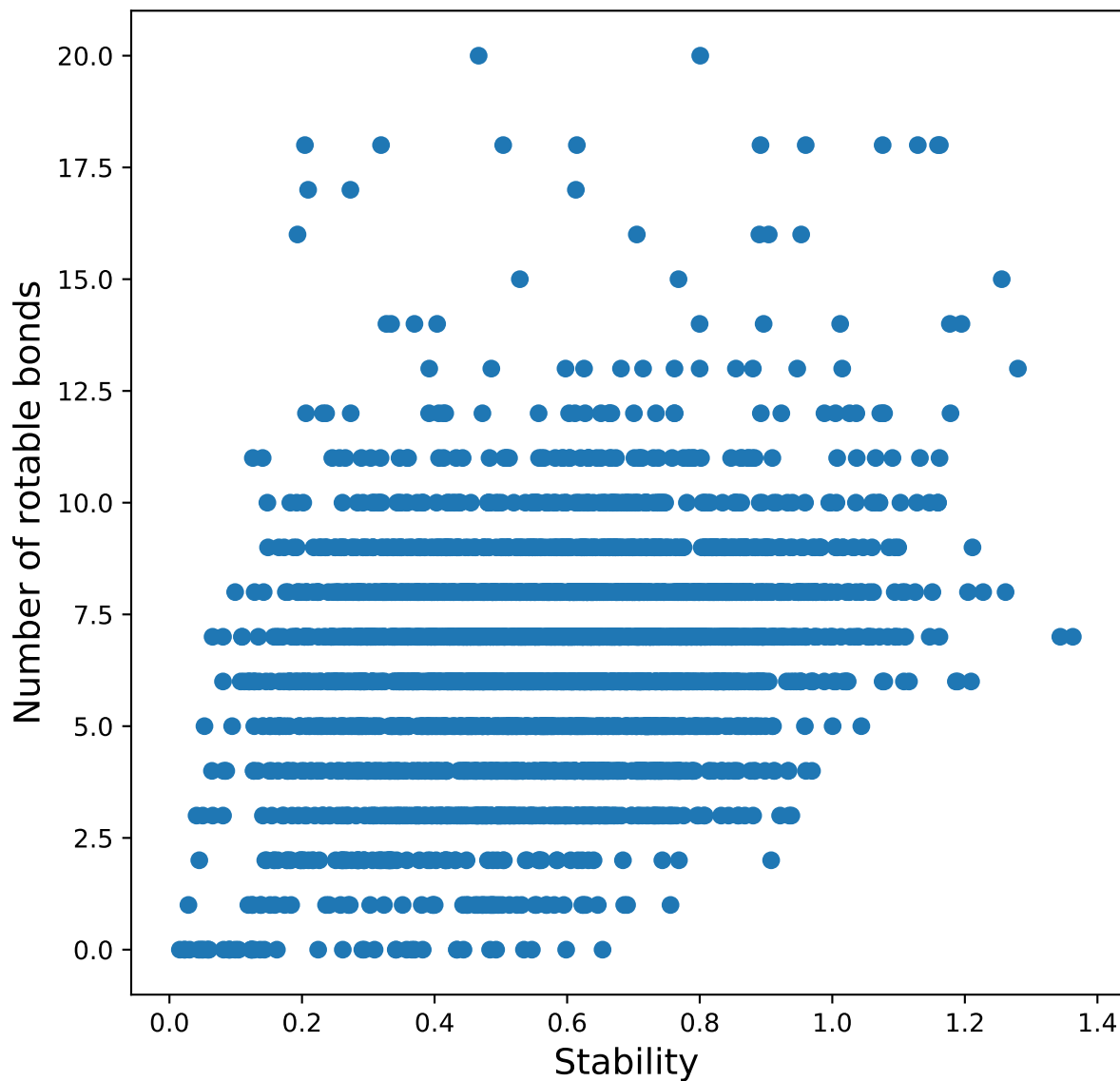

Supplement: Supplementary file 2 — Visualization of the correlation between the number of rotatable bonds and the variations in atom positions in the docked poses (ZIP 1316 kb) [file 11030_2018_9894_MOESM2_ESM.zip › Supplementary_File_S2/stability_pose_K=3_D3.pdf]

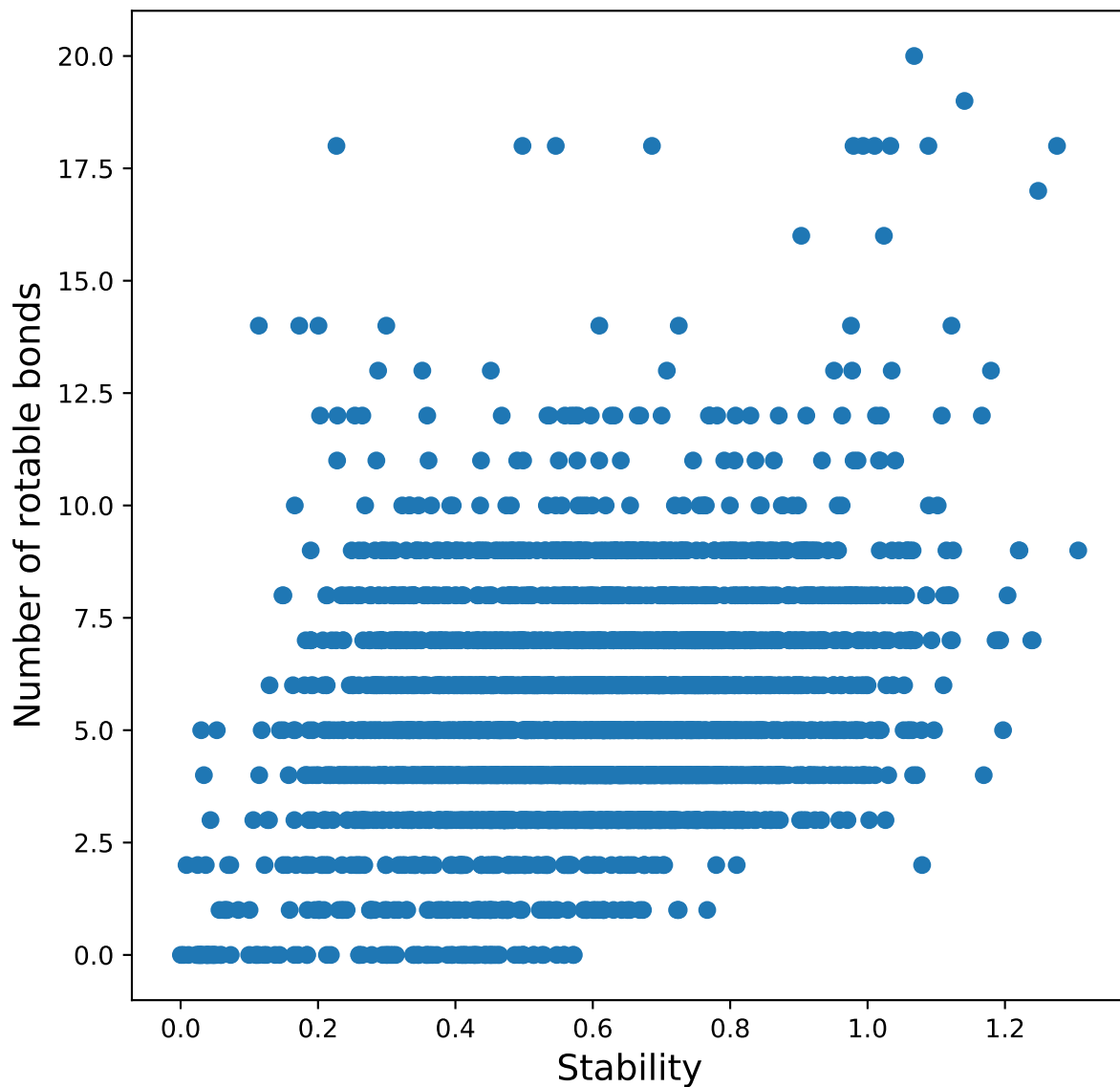

Supplement: Supplementary file 2 — Visualization of the correlation between the number of rotatable bonds and the variations in atom positions in the docked poses (ZIP 1316 kb) [file 11030_2018_9894_MOESM2_ESM.zip › Supplementary_File_S2/stability_pose_K=3_D4.pdf]

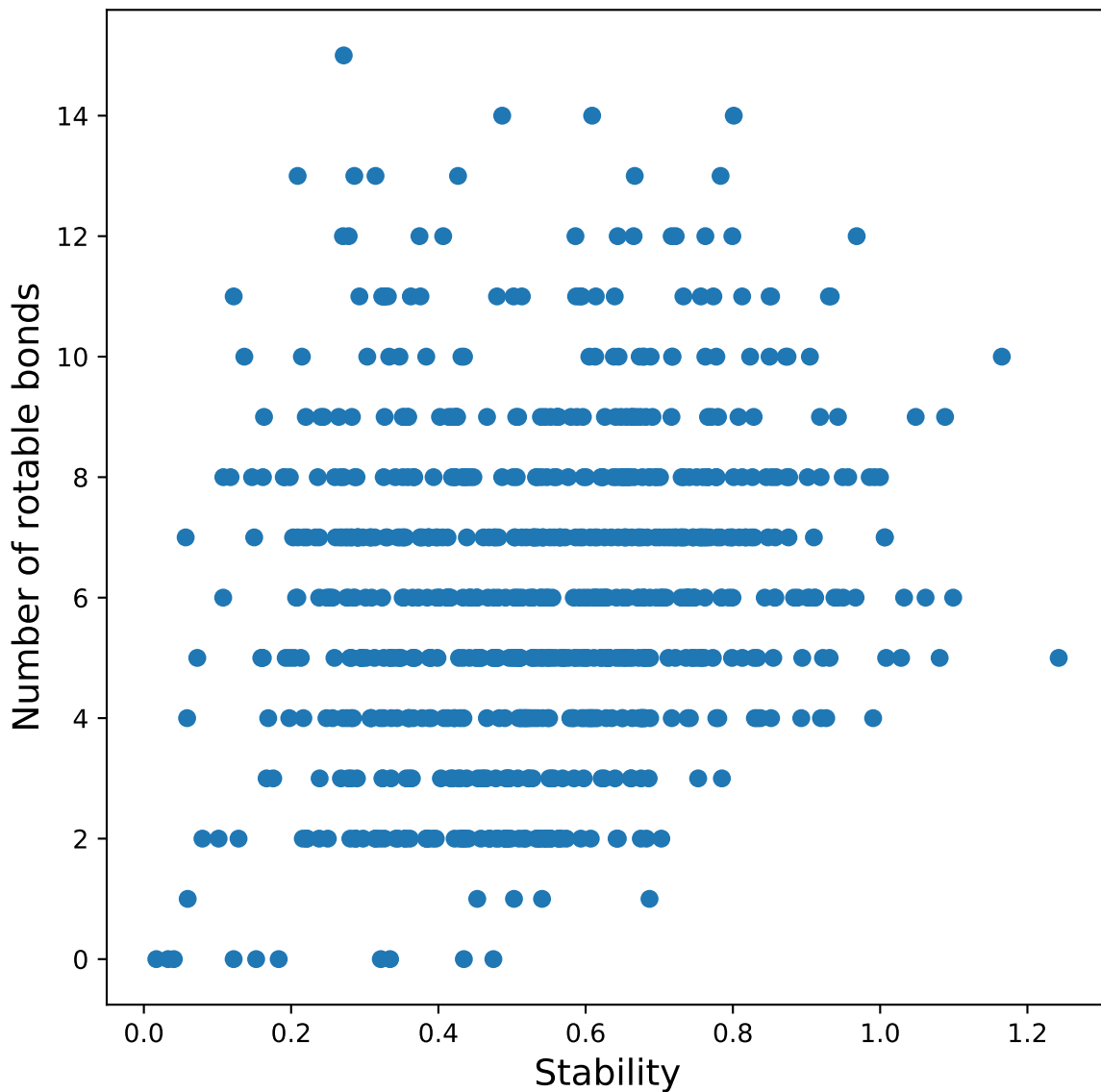

Supplement: Supplementary file 2 — Visualization of the correlation between the number of rotatable bonds and the variations in atom positions in the docked poses (ZIP 1316 kb) [file 11030_2018_9894_MOESM2_ESM.zip › Supplementary_File_S2/stability_pose_K=3_H1.pdf]

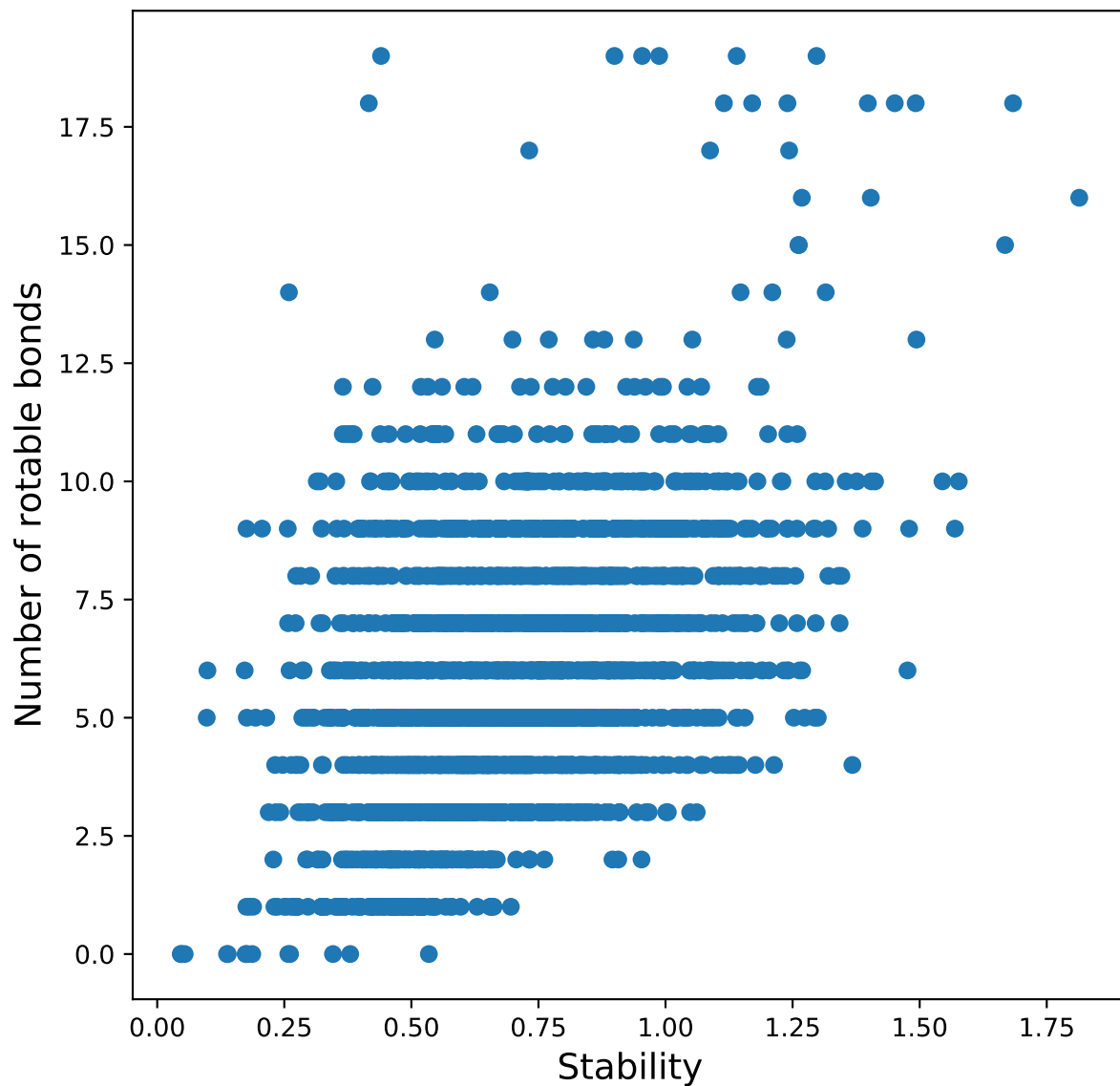

Supplement: Supplementary file 2 — Visualization of the correlation between the number of rotatable bonds and the variations in atom positions in the docked poses (ZIP 1316 kb) [file 11030_2018_9894_MOESM2_ESM.zip › Supplementary_File_S2/stability_pose_K=5_5HT1B.pdf]

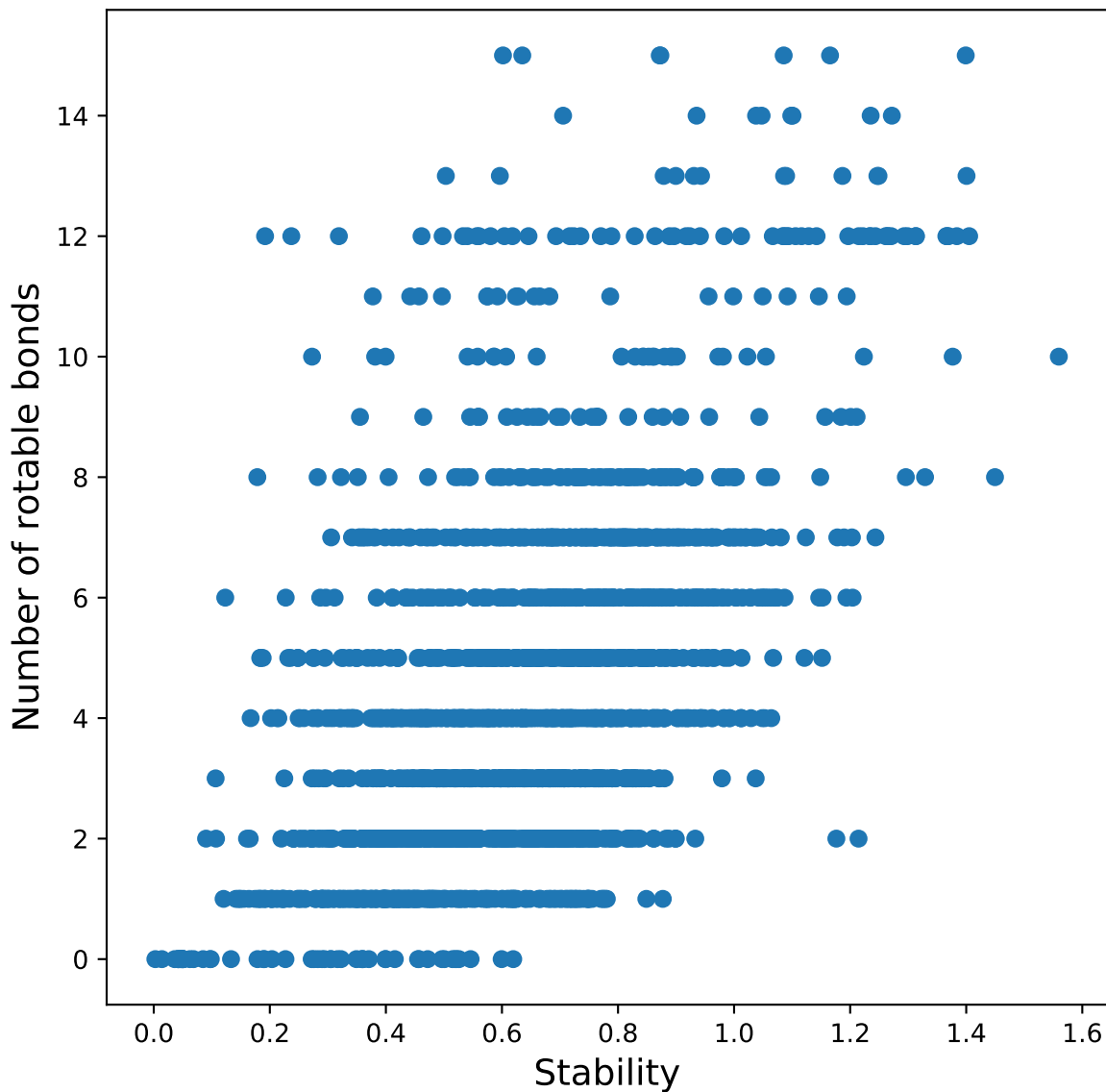

Supplement: Supplementary file 2 — Visualization of the correlation between the number of rotatable bonds and the variations in atom positions in the docked poses (ZIP 1316 kb) [file 11030_2018_9894_MOESM2_ESM.zip › Supplementary_File_S2/stability_pose_K=5_5HT2B.pdf]

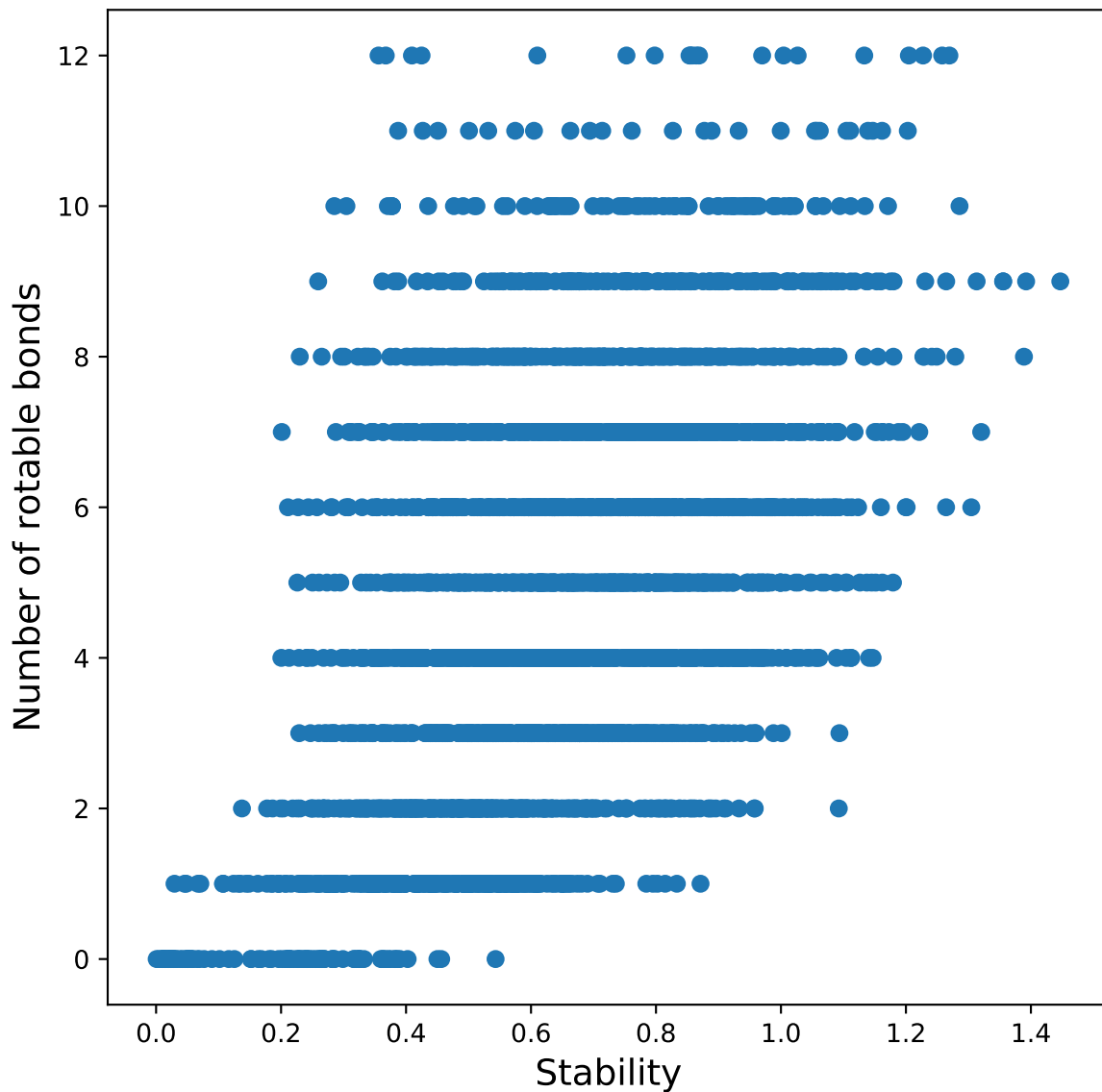

Supplement: Supplementary file 2 — Visualization of the correlation between the number of rotatable bonds and the variations in atom positions in the docked poses (ZIP 1316 kb) [file 11030_2018_9894_MOESM2_ESM.zip › Supplementary_File_S2/stability_pose_K=5_5HT2C.pdf]

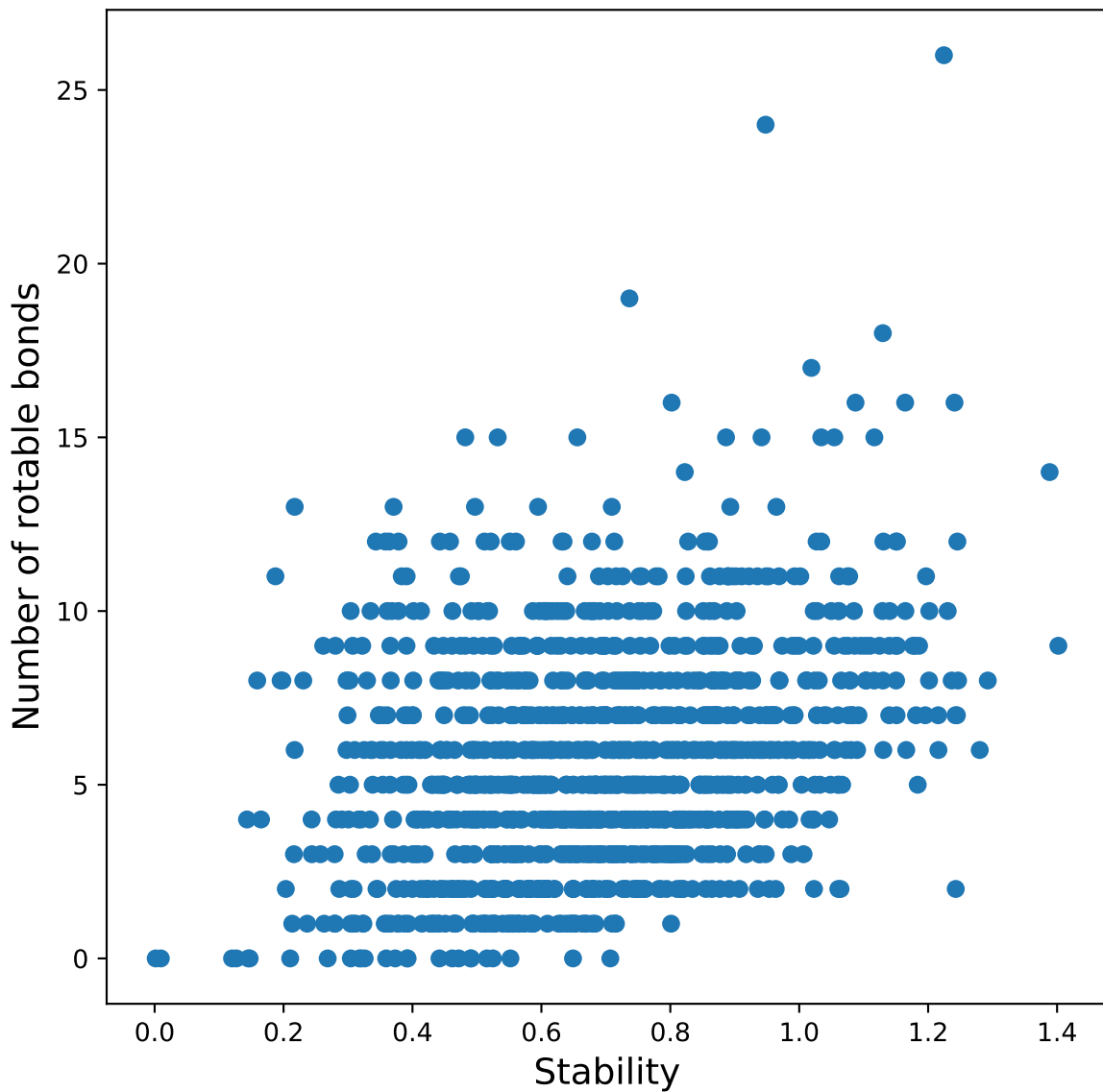

Supplement: Supplementary file 2 — Visualization of the correlation between the number of rotatable bonds and the variations in atom positions in the docked poses (ZIP 1316 kb) [file 11030_2018_9894_MOESM2_ESM.zip › Supplementary_File_S2/stability_pose_K=5_ACM1.pdf]

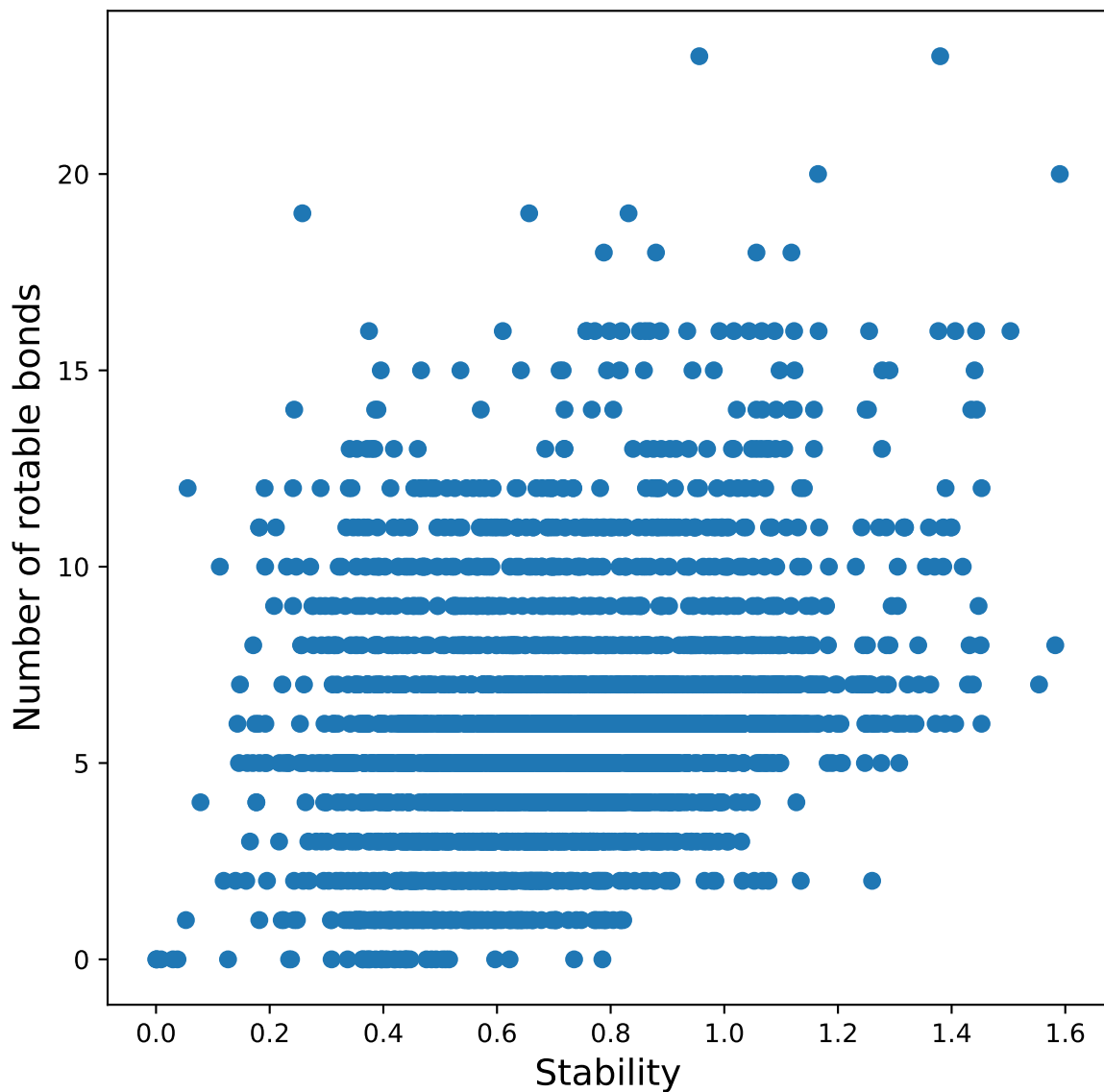

Supplement: Supplementary file 2 — Visualization of the correlation between the number of rotatable bonds and the variations in atom positions in the docked poses (ZIP 1316 kb) [file 11030_2018_9894_MOESM2_ESM.zip › Supplementary_File_S2/stability_pose_K=5_ACM2.pdf]

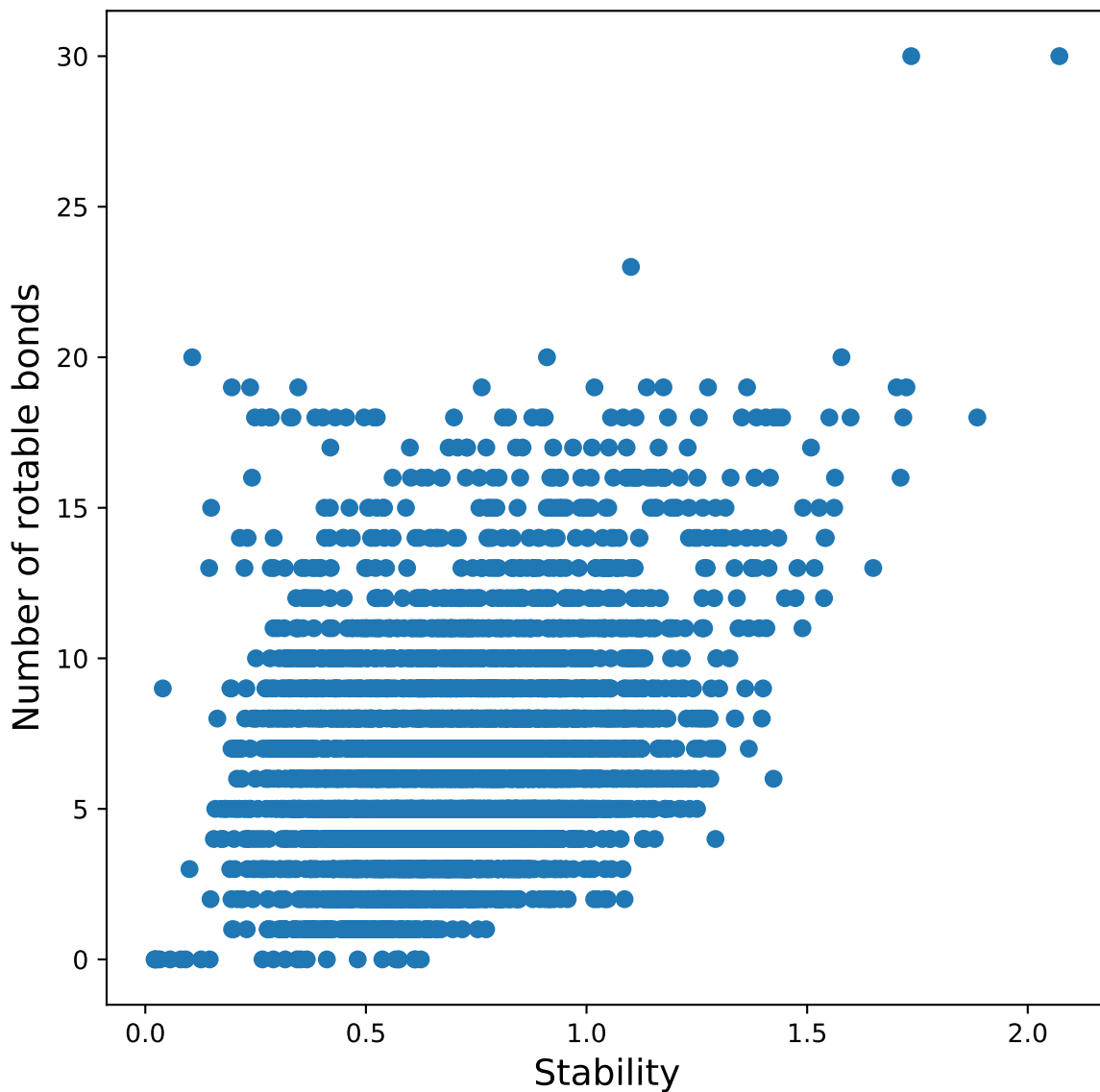

Supplement: Supplementary file 2 — Visualization of the correlation between the number of rotatable bonds and the variations in atom positions in the docked poses (ZIP 1316 kb) [file 11030_2018_9894_MOESM2_ESM.zip › Supplementary_File_S2/stability_pose_K=5_ACM3.pdf]

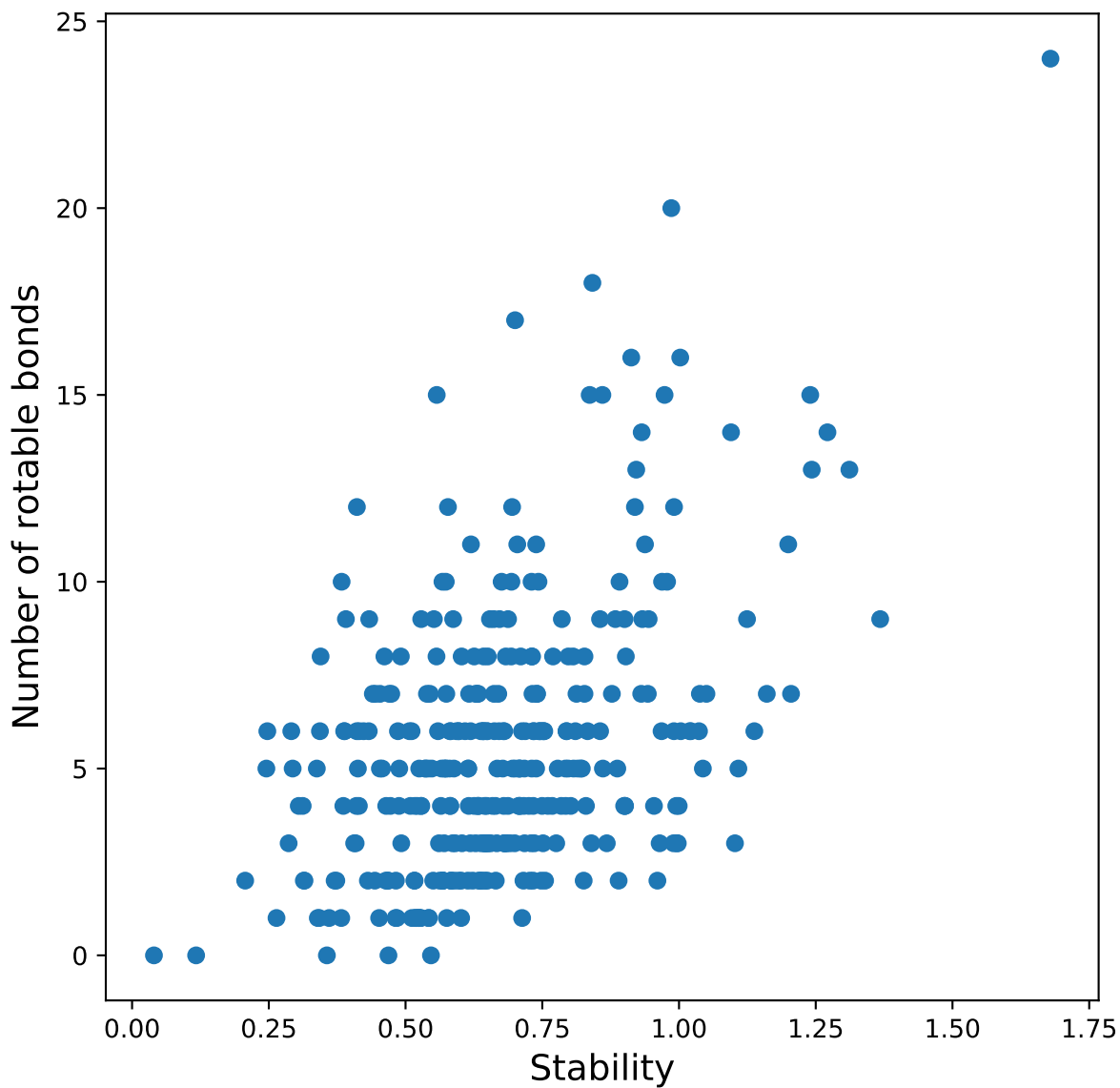

Supplement: Supplementary file 2 — Visualization of the correlation between the number of rotatable bonds and the variations in atom positions in the docked poses (ZIP 1316 kb) [file 11030_2018_9894_MOESM2_ESM.zip › Supplementary_File_S2/stability_pose_K=5_ACM4.pdf]

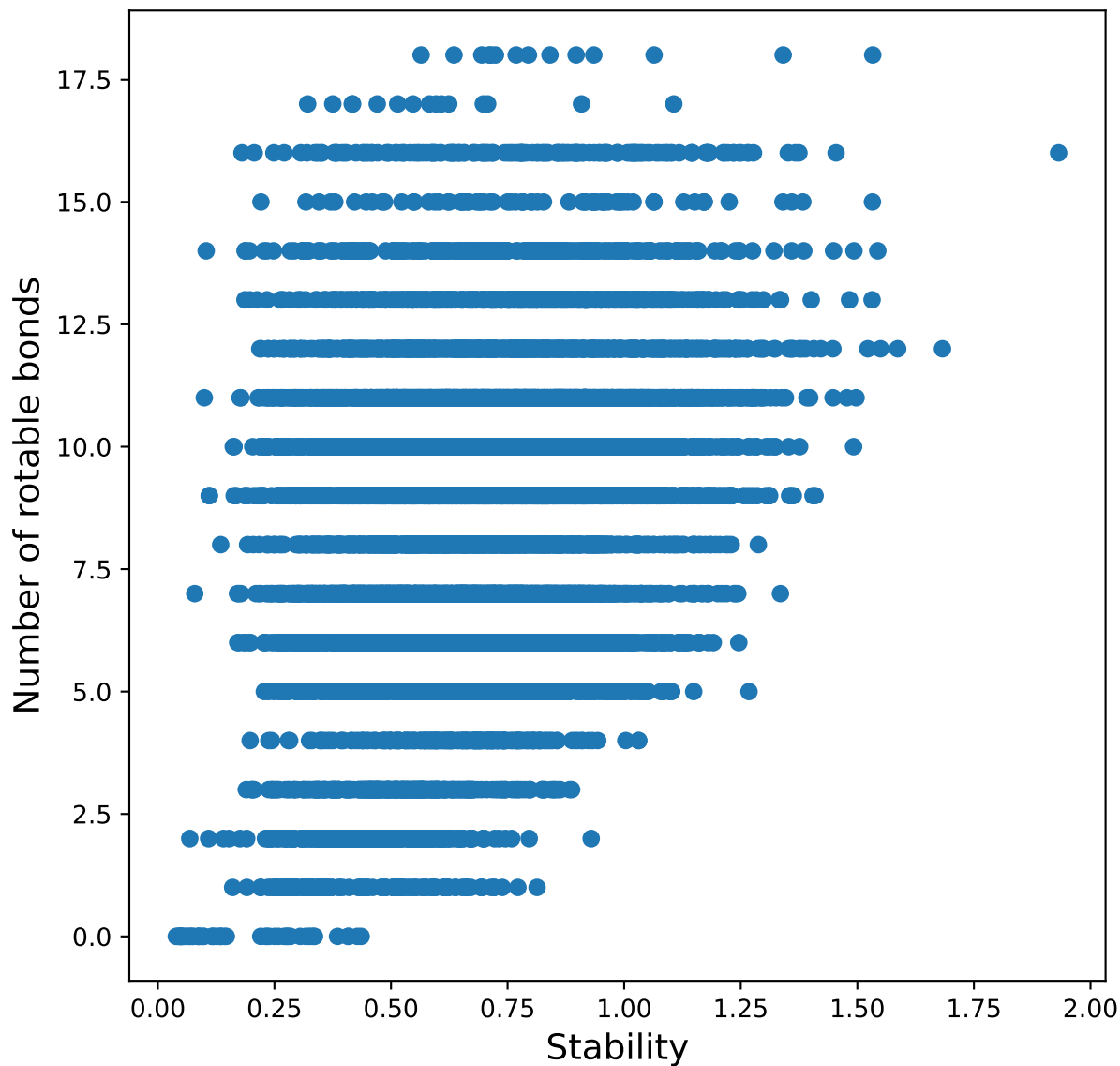

Supplement: Supplementary file 2 — Visualization of the correlation between the number of rotatable bonds and the variations in atom positions in the docked poses (ZIP 1316 kb) [file 11030_2018_9894_MOESM2_ESM.zip › Supplementary_File_S2/stability_pose_K=5_BETA1.pdf]

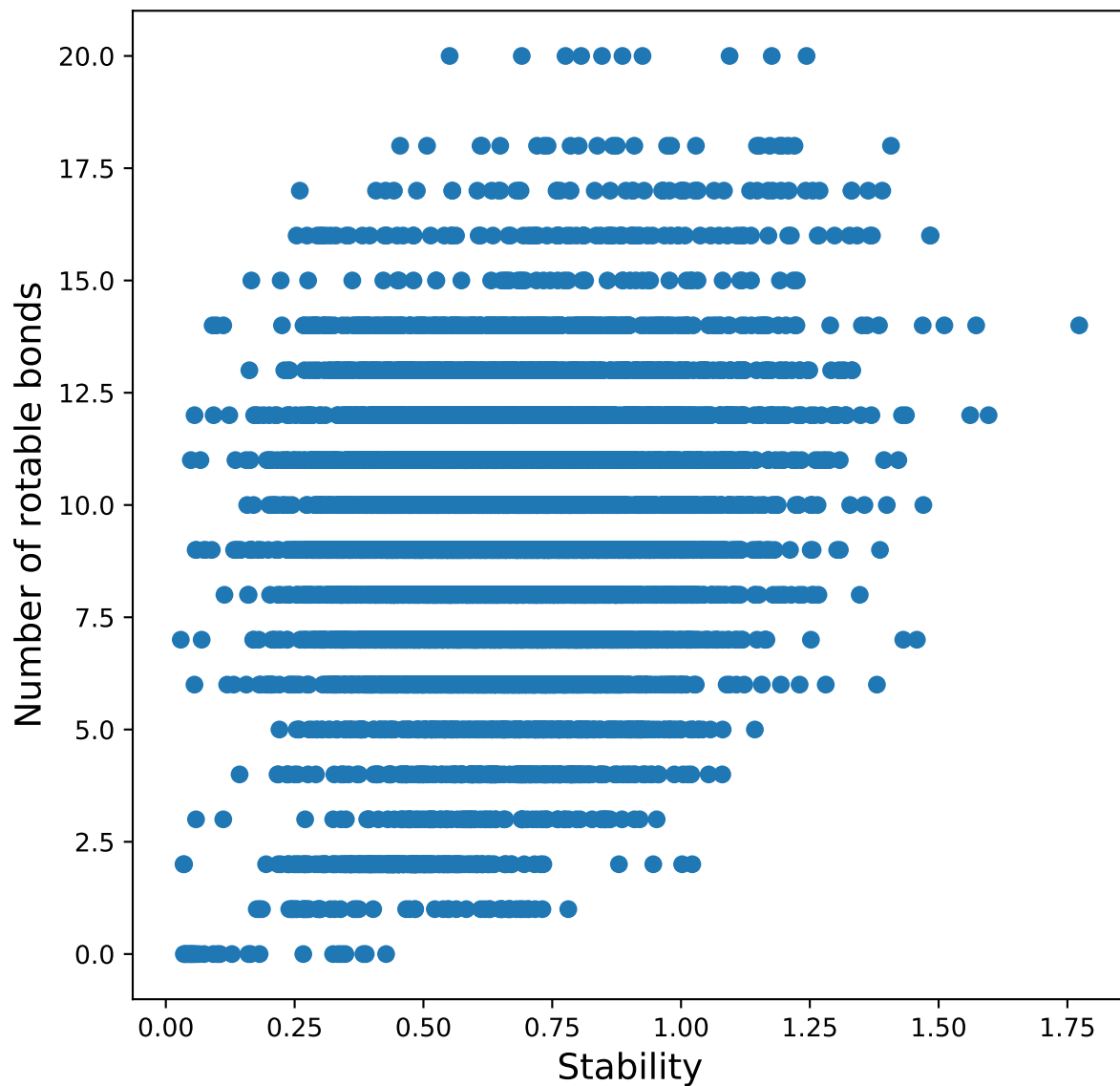

Supplement: Supplementary file 2 — Visualization of the correlation between the number of rotatable bonds and the variations in atom positions in the docked poses (ZIP 1316 kb) [file 11030_2018_9894_MOESM2_ESM.zip › Supplementary_File_S2/stability_pose_K=5_BETA2.pdf]

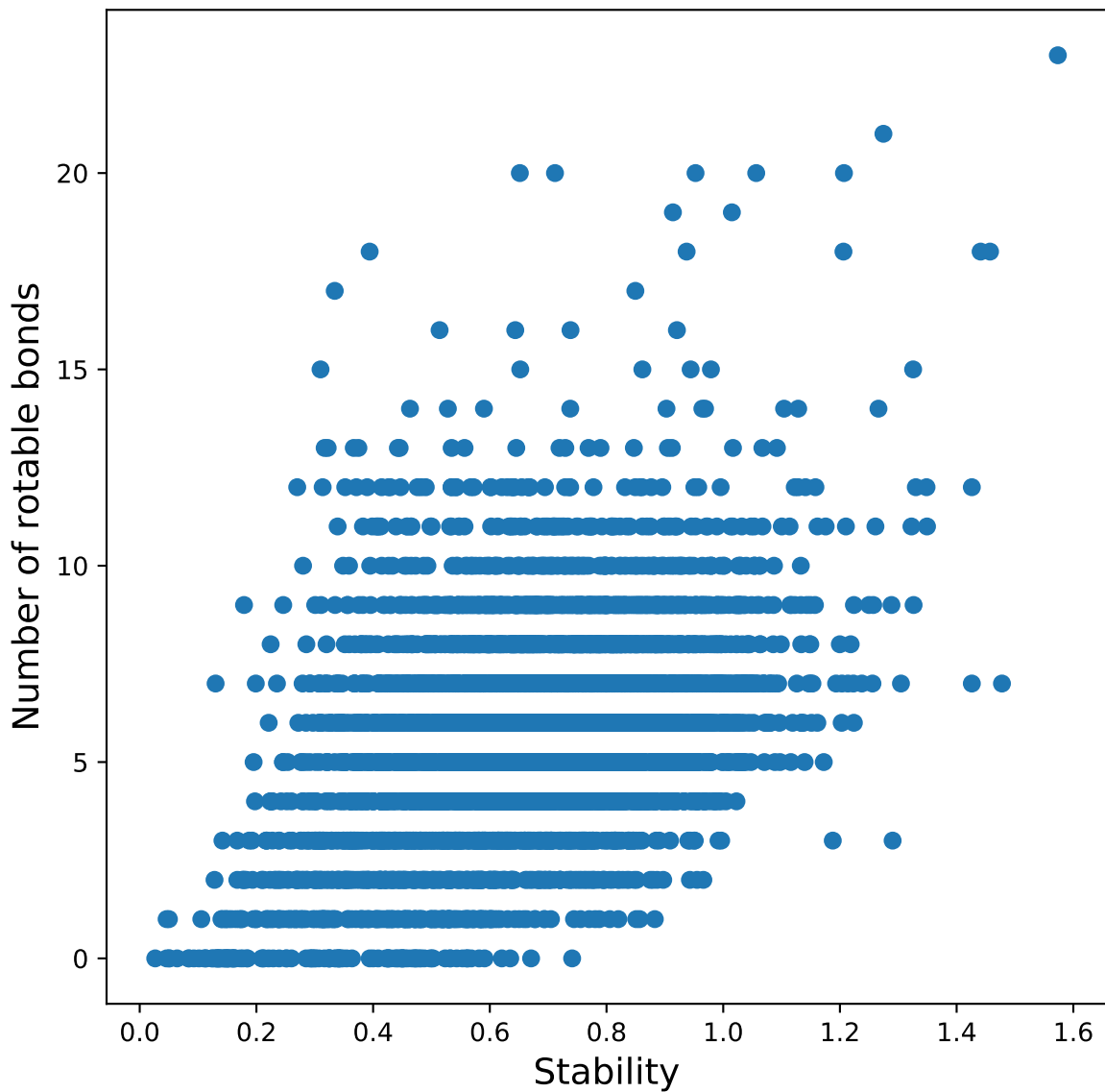

Supplement: Supplementary file 2 — Visualization of the correlation between the number of rotatable bonds and the variations in atom positions in the docked poses (ZIP 1316 kb) [file 11030_2018_9894_MOESM2_ESM.zip › Supplementary_File_S2/stability_pose_K=5_D2.pdf]

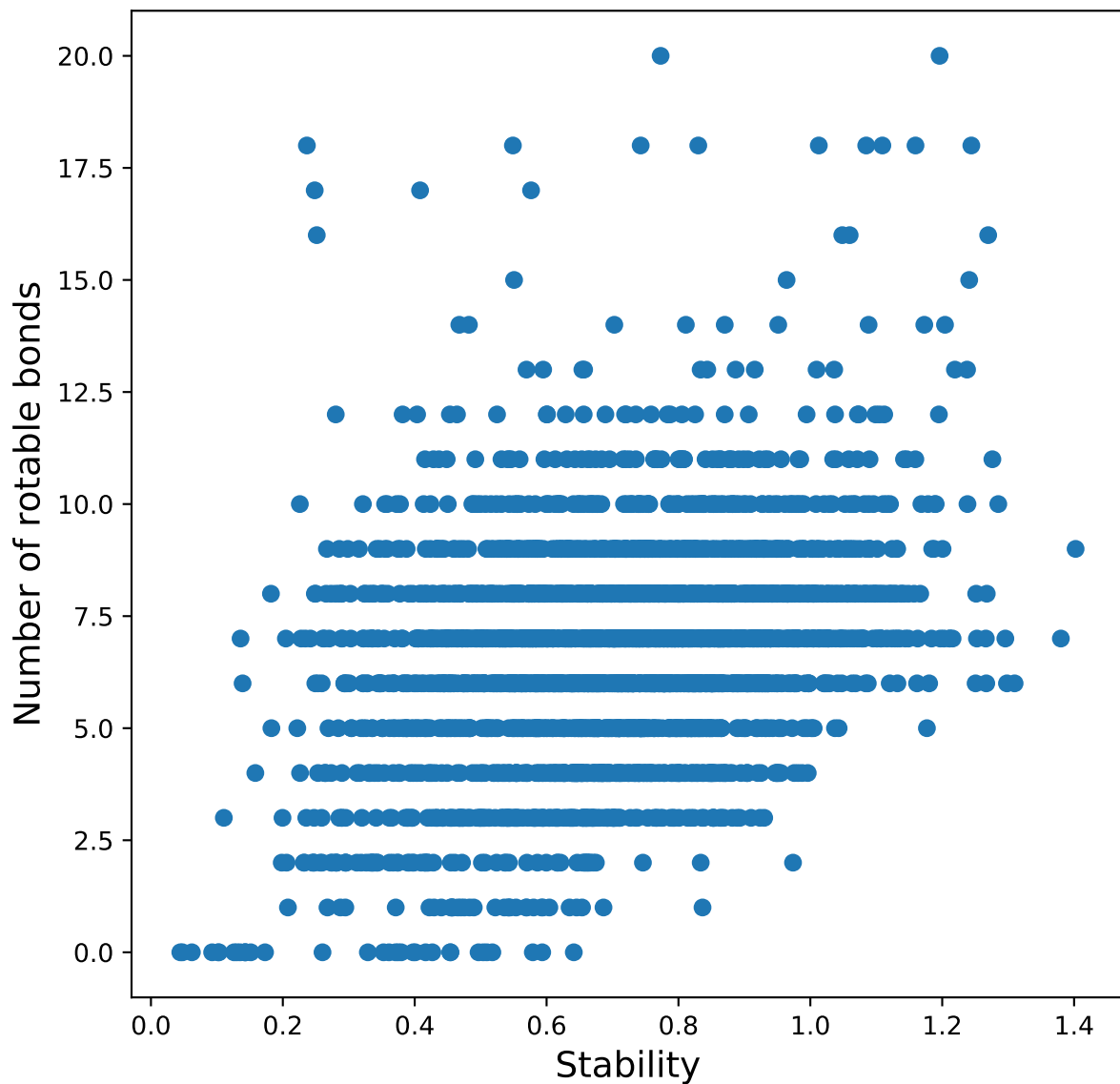

Supplement: Supplementary file 2 — Visualization of the correlation between the number of rotatable bonds and the variations in atom positions in the docked poses (ZIP 1316 kb) [file 11030_2018_9894_MOESM2_ESM.zip › Supplementary_File_S2/stability_pose_K=5_D3.pdf]

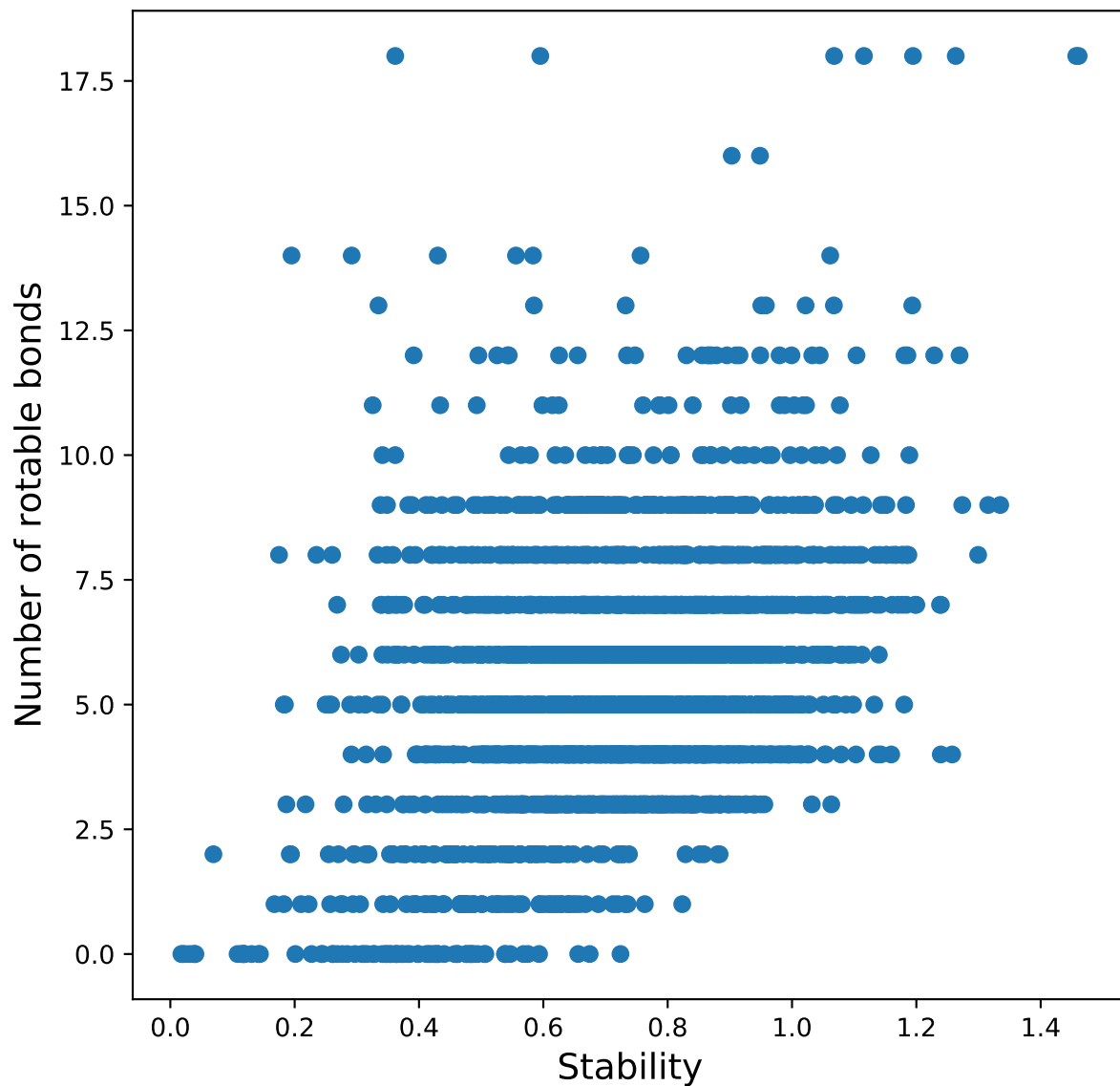

Supplement: Supplementary file 2 — Visualization of the correlation between the number of rotatable bonds and the variations in atom positions in the docked poses (ZIP 1316 kb) [file 11030_2018_9894_MOESM2_ESM.zip › Supplementary_File_S2/stability_pose_K=5_D4.pdf]

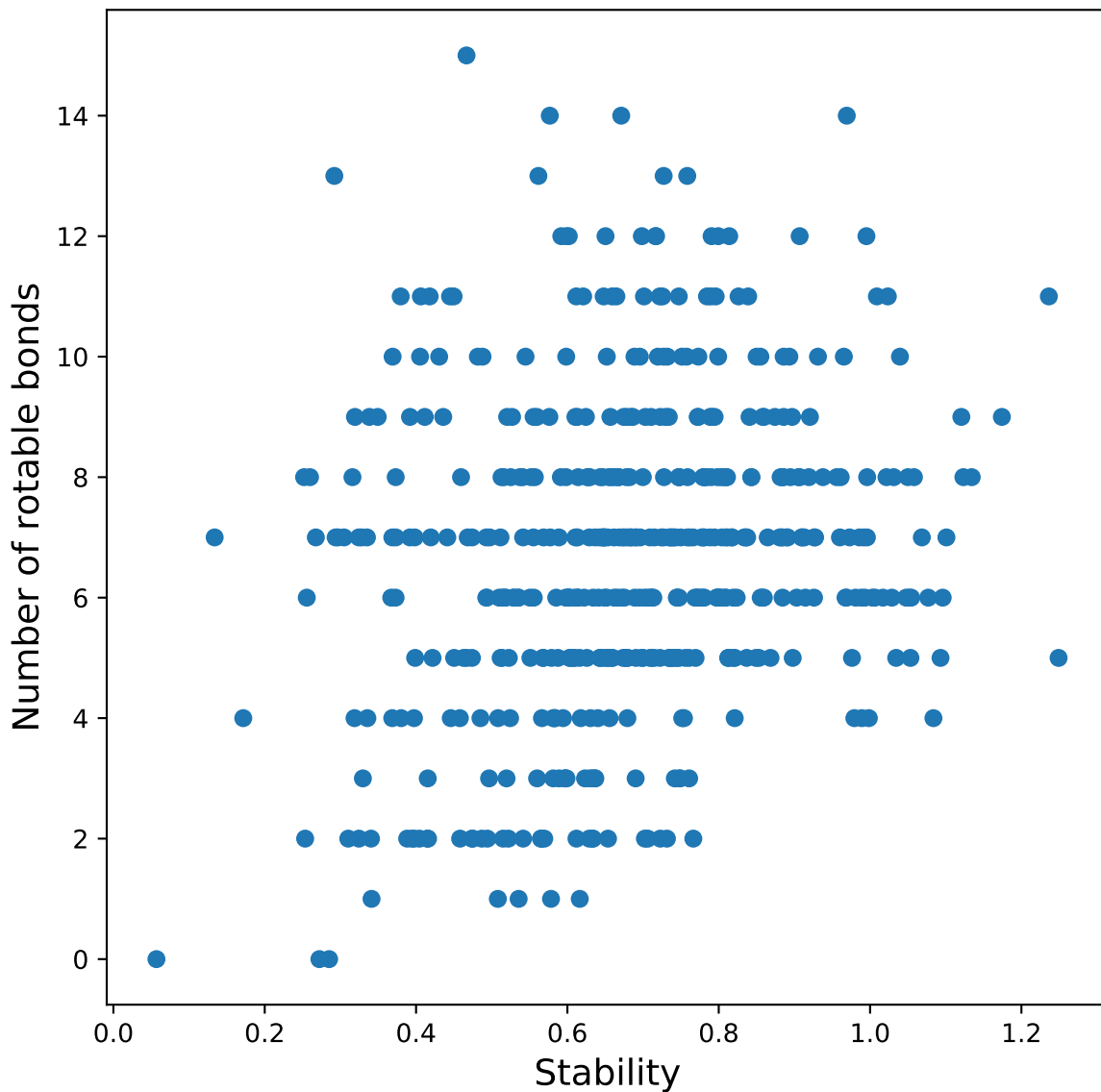

Supplement: Supplementary file 2 — Visualization of the correlation between the number of rotatable bonds and the variations in atom positions in the docked poses (ZIP 1316 kb) [file 11030_2018_9894_MOESM2_ESM.zip › Supplementary_File_S2/stability_pose_K=5_H1.pdf]
